# Host-linked soil viral ecology along a permafrost thaw gradient

**DOI:** 10.1038/s41564-018-0190-y

**Published:** 2018-07-16

**Authors:** Joanne B. Emerson, Simon Roux, Jennifer R. Brum, Benjamin Bolduc, Ben J. Woodcroft, Ho Bin Jang, Caitlin M. Singleton, Lindsey M. Solden, Adrian E. Naas, Joel A. Boyd, Suzanne B. Hodgkins, Rachel M. Wilson, Gareth Trubl, Changsheng Li, Steve Frolking, Phillip B. Pope, Kelly C. Wrighton, Patrick M. Crill, Jeffrey P. Chanton, Scott R. Saleska, Gene W. Tyson, Virginia I. Rich, Matthew B. Sullivan

**Affiliations:** 1 Department of Microbiology, The Ohio State University, Columbus, OH USA; 2 Australian Centre for Ecogenomics, School of Chemistry and Molecular Biosciences, University of Queensland, Brisbane, Brisbane, Queensland Australia; 3 Faculty of Chemistry, Biotechnology and Food Science, Norwegian University of Life Sciences, Ås, Norway; 4 Department of Earth, Ocean, and Atmospheric Science, Florida State University, Tallahassee, FL USA; 5 Earth Systems Research Center, Institute for the Study of Earth, Oceans and Space, University of New Hampshire, Durham, NH USA; 6 Department of Geological Sciences, Stockholm University, Stockholm, Sweden; 7 Department of Ecology and Evolutionary Biology, University of Arizona, Tucson, AZ USA; 8 Department of Civil, Environmental and Geodetic Engineering, The Ohio State University, Columbus, OH USA; 9 Present Address: Department of Plant Pathology, University of California, Davis, Davis, CA USA; 10 Present Address: United States Department of Energy Joint Genome Institute, Lawrence Berkeley National Laboratory, Walnut Creek, CA USA; 11 Present Address: Louisiana State University, Baton Rouge, LA USA

**Keywords:** Metagenomics, Microbial communities, Microbial ecology

## Abstract

Climate change threatens to release abundant carbon that is sequestered at high latitudes, but the constraints on microbial metabolisms that mediate the release of methane and carbon dioxide are poorly understood^1–7^. The role of viruses, which are known to affect microbial dynamics, metabolism and biogeochemistry in the oceans^8–10^, remains largely unexplored in soil. Here, we aimed to investigate how viruses influence microbial ecology and carbon metabolism in peatland soils along a permafrost thaw gradient in Sweden. We recovered 1,907 viral populations (genomes and large genome fragments) from 197 bulk soil and size-fractionated metagenomes, 58% of which were detected in metatranscriptomes and presumed to be active. In silico predictions linked 35% of the viruses to microbial host populations, highlighting likely viral predators of key carbon-cycling microorganisms, including methanogens and methanotrophs. Lineage-specific virus/host ratios varied, suggesting that viral infection dynamics may differentially impact microbial responses to a changing climate. Virus-encoded glycoside hydrolases, including an endomannanase with confirmed functional activity, indicated that viruses influence complex carbon degradation and that viral abundances were significant predictors of methane dynamics. These findings suggest that viruses may impact ecosystem function in climate-critical, terrestrial habitats and identify multiple potential viral contributions to soil carbon cycling.

## Abstract (web summary)

The recovery of viral populations from peatland soils across a permafrost thaw gradient provides insights into soil viral diversity, their hosts and the potential impacts on carbon cycling in this environment.

## Main

Thawing permafrost soils are expected to emit substantial microbially generated methane (CH_4_) and carbon dioxide (CO_2_)^1,4^, but the magnitude of the resulting positive feedback to climate is poorly constrained^11,12^. Microbial ecological studies may provide a basis for improving the predictions of these shifting outputs^6,13–15^. Although viral ecology is largely unexplored in soils, viruses lyse approximately one-third of ocean microorganisms per day, can metabolically reprogramme their hosts during infection and act as agents of horizontal gene transfer^8,10^. These viral effects substantially impact ecosystem processes; compared to prokaryotic and eukaryotic microbial abundances, viral population abundances best predicted the global carbon flux from the surface oceans to the deep sea^9^.

Although several pioneering soil viral metagenomic efforts have been reported^16^, genome-enabled approaches to soil viral ecology have only recently emerged. Recent advances enable viral purification from peatland soils for metagenomics^17^, as well as mining viral sequences from complex microbial (meta)genomic data sets in silico^18,19^. Here, we recovered viral genomes from bulk and size-fractionated peatland soil metagenomes and assessed their potential ecological and biogeochemical impacts along a permafrost thaw gradient. Samples were collected from the thawed, active surface layer of three habitats (in order of increasing thaw: palsa, bog and fen) in Stordalen Mire (Supplementary Fig. 1), a long-term climate change research site in northern Sweden^2,3,6^.

**Fig. 1:**
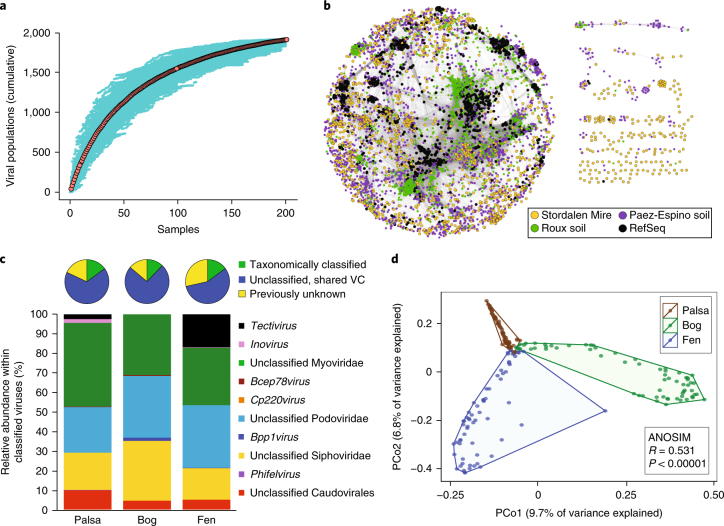
Overview of Stordalen Mire soil viruses. **a**, An accumulation curve of viral populations in bulk soil metagenomes (*n* = 201). The means are represented by red circles and 200 randomizations of sample order are shown in teal. **b**, A network of shared predicted protein content among Stordalen Mire viruses (*n* = 1,907), RefSeq prokaryotic viral genomes (*n* = 2,010) and soil-associated viral contigs >10 kb from Paez-Espino et al.^19^ (*n* = 3,112) and Roux et al.^18^ (*n* = 2,040). Nodes (circles) represent genomes and contigs, and the shared edges (lines) indicate shared protein content. **c**, Pie charts indicate per cent relative abundances of Stordalen Mire viral populations (*n* = 828, 782 and 475 populations detected in palsa, bog and fen, respectively; palsa: *n* = 72 samples, bog: *n* = 65 samples and fen: *n* = 64 samples) that: have predicted taxonomy (green), have unknown taxonomy but share a viral cluster (VC) with viruses from public datasets (from **b**, blue), or were previously unknown (in a Stordalen Mire-exclusive VC, yellow). The bar graphs indicate the per cent relative abundances of viral taxa in each habitat, considering only viruses with predicted taxonomy (*n* = 323). **d**, Principal coordinates analysis (PCoA) of viral community composition, as derived from read mapping to viral contigs (*n* = 1,907) and Bray–Curtis dissimilarities; each point is one sample (*n* = 201). The analysis of similarity (ANOSIM) statistics consider viral community composition grouped by habitat (palsa: *n* = 72 samples, bog: *n* = 65 samples and fen: *n* = 64 samples).

Assemblies from 178 bulk soil metagenomes (collected from 2010–2012)^14^, 12 size-fractionated metagenomes (collected in 2014 for the enrichment of small microorganisms) and 7 viromes (collected in 2014), all from Stordalen soils, were screened using VirSorter^18^ and manually curated (Supplementary Fig. 2 and Supplementary Table 1) to recover 7,547 viral contigs. These Stordalen contigs were combined with 15,220 viral genomes and large genome fragments from publicly available data sets (RefSeq prokaryotic viral genomes and viral genomes mined from microbial genomes^18,20^) for a total of 22,767 viral contigs, which were clustered at 95% nucleotide identity to define 17,434 viral ‘populations’ that approximately represent species-level taxonomy^21^. To maximize the recovery of viral populations from 214 Stordalen bulk soil metagenomes^14^, we used this initial database of viral population sequences for detection through read mapping (Supplementary Fig. 2). A final set of 1,907 viral populations was detected in 201 of the 214 bulk soil metagenomes and used in downstream analyses. These populations are represented by contigs ≥10 kb in size and/or circular (assumed to be complete), and all of the detected populations were assembled from Stordalen metagenomes; no viruses from the considered public databases were detected. Because these viral populations were detected in bulk soil DNA, they presumably represent free viruses, proviruses (that is, integrated or extrachromosomal viruses that replicate with their hosts) and/or actively infecting viruses. The viral communities were relatively well sampled across all three habitats (Fig. 1a and Supplementary Fig. 3), and 1,106 (58%) of the viral populations were detected in metatranscriptomes (generated from 21 of the 201 bulk soil samples), suggesting that many of the viral populations were active in this system (Supplementary Table 2).

To investigate the similarity of Stordalen viral populations to relevant, publicly available sequences and assign them taxonomy, we used a genome-based network analysis of their shared protein content^22^ with three data sets: (1) 2,010 prokaryotic viral genomes (RefSeq v75^20^), (2) 2,040 soil viral contigs mined from isolate microbial genomes^18^ and (3) 3,112 soil-associated viral contigs recovered from bulk metagenomes^19^. This analysis grouped viral populations at approximately the genus level into viral clusters^18,22^. In the network, viral populations that were derived from microbial isolates generally formed more cohesive clusters than did viral populations recovered from metagenomes (Fig. 1b), suggesting that culture-based studies do not adequately capture soil viral diversity. Across the four data sets, 1,972 viral clusters were identified, of which 1,851 (94%) contained uncultivated soil viral sequences from Stordalen Mire and/or the other two bioinformatically mined data sets^18,19^ (Supplementary Table 3). Stordalen viral populations were found in 738 viral clusters, 451 of which were Stordalen exclusive, more than doubling the number of prokaryotic viral genera in the RefSeq database (306 viral clusters; Supplementary Fig. 4). Only 17% of the Stordalen viral populations could be assigned taxonomy and most of those (95%) were only coarsely resolved as unclassified members of the *Caudovirales* order and its families (Fig. 1c). These findings reveal substantial previously unknown genomic and taxonomic diversity in soil viral communities, which have been poorly sampled to date^16,19,23,24^. In addition, ~38% of the Stordalen viral clusters were present in at least one other soil-associated data set, indicating some sequence conservation across globally distributed soils and suggesting that soil viral communities may have some broad biogeographical patterns in common with aqueous systems^25–27^.

Based on read mapping from 72 palsa, 65 bog and 64 fen (201 total) metagenomes and 4 palsa, 7 bog and 10 fen (21 total) metatranscriptomes, respectively, to the 1,907 viral populations, both viral and ‘active’ viral community composition differed significantly along the thaw gradient (Fig. 1d and Supplementary Fig. 5). Together with significant differences in within-habitat variance (using a homogeneity of dispersions test, PERMDISP *F* = 25.8, *P* < 0.0001), these results indicate that viral communities may change as permafrost continues to thaw, paralleling known shifts in bacterial and archaeal community composition, environmental conditions, vegetation and organic matter composition^2,6,14^. Mantel tests revealed that viral community composition significantly correlated with host community composition and, consistent with known patterns in bacterial community composition^28^, with pH and soil moisture content (Supplementary Table 4). Within habitats, viral communities were characterized by high endemism (Supplementary Fig. 6) and were structured by soil depth (Supplementary Fig. 7).

**Fig. 2:**
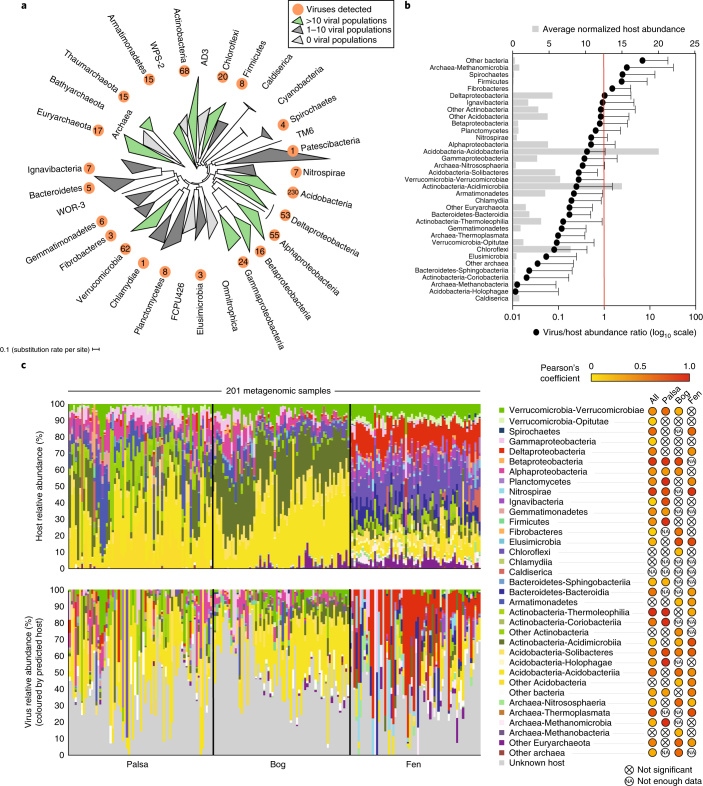
Stordalen Mire virus–host linkages and abundance patterns. **a**, Phylogenetic tree of bacterial and archaeal phyla (classes for Proteobacteria) with population genomes recovered from Stordalen Mire. The tree was constructed from concatenated protein sequences of single-copy genes, as in ref. ^14^. The orange circles indicate the lineages predicted to be infected by viruses, with the number of viruses shown within the circle. **b**, Virus/host abundance ratios by host lineage (bottom *x*-axis), calculated as the ratio of per base-pair average coverage depth from read mapping to viral contigs and host population genomes, respectively, normalized by the number of sequencing reads in each sample. The dots indicate the mean ratio across samples (*n* = 201), and the error bars indicate one standard deviation. The red line indicates the 1/1 virus/host abundance ratio. The normalized host abundance (top *x*-axis) is an average across all samples. **c**, Host and virus abundance, grouped by predicted host taxonomy. The host and virus relative abundances across the Stordalen Mire bulk soil metagenomes (*n* = 201), based on read mapping, are shown. Samples are separated by habitat (black vertical lines), and, within each habitat, are ordered by depth; within the depth regimes, samples are in chronological order of sampling date. Of the 140 virus–host pairs tested, Pearson’s correlation coefficients from 75 significantly correlated abundances (*P* < 0.05) are coloured according to the key (values appear in Supplementary Table 10); the probability of observing only 13 or more such *P* < 0.05 correlations given the 140 tests is less than 5% under the null hypothesis.

We next investigated whether soil viruses, like some of their marine counterparts^8,10,25^, might affect ecosystem function by infecting microorganisms that drive biogeochemical cycles. We screened a database of 1,529 Stordalen bacterial and archaeal population genomes^14^, recovered largely from the same metagenomes as the viral populations, for genomic features to link viruses to hosts^25,29^. Hosts were predicted for 667 (35%) of the viral populations (Supplementary Table 5), a nearly 5-fold increase over the percentage of viruses (7.7%) linked to hosts in a recent global study^19^, illustrating the benefit of reconstructing host and viral genomes from the same samples. Stordalen viruses were linked to hosts from 19 bacterial and archaeal phyla (Fig. 2a) and included 15 viruses of methanogens, 13 methanotroph viruses and numerous viruses of respiring and fermentative heterotrophs^14^ (Supplementary Tables 6 and 7). These results suggest that viruses have the potential to indirectly (through mortality of key microbial carbon cyclers) affect carbon cycling in Stordalen Mire.

**Fig. 3:**
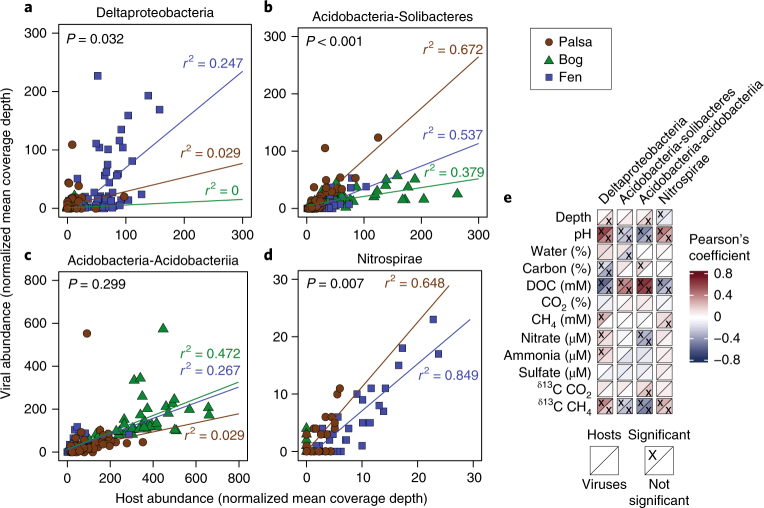
Examples of lineage-specific virus–host abundance patterns. **a**–**d**, Virus/host abundance ratios for specific host lineages, indicated at the top of each plot (palsa: *n* = 72, bog: *n* = 65 and fen: *n* = 64). Host lineage abundance and the abundance of viruses for that host (both calculated as the mean coverage depth from metagenomic read mapping, normalized by the number of reads in the sample) are plotted for each sample in which viruses and/or hosts were detected. Note the different axis maxima among graphs. Based on linear regression analysis (Supplementary Table 9), colour-coded best-fit lines and adjusted *r*^2^ values for each habitat are presented (there was not enough data for the bog habitat in panel **d**). ANOVA *P* values (999 permutations, significant when *P* < 0.05) indicate whether the interaction term in the linear regression models (that is, sample designations as palsa, bog or fen) was significantly different from not using an interaction term (that is, all samples together). **e**, Pearson’s correlation coefficients for the four host lineages from **a**–**d** and their viruses, correlated with environmental and geochemical measurements (significant when *P* < 0.05; values appear in Supplementary Table 12); 38 significant correlations are depicted with an ‘x’. The probability of observing only 9 or more such *P* < 0.05 correlations given 96 tests is less than 5% under the null hypothesis.

To further investigate potential viral effects on host ecology, we assessed how viral infection dynamics for specific host lineages varied across the three permafrost thaw habitats. Lineage-specific virus/host abundance ratios (from read mapping of 201 bulk soil metagenomes to the 1,907 viral and 1,529 host population sequences) were used for these analyses, grouped at the host class or phylum level (Fig. 2b and Supplementary Tables 7 and 8). Among lineages, a range of virus/host abundance ratios was observed, with average viral abundances often close to or below host abundances (Fig. 2b). Most (22 of the 35) lineage-specific virus/host abundance relationships differed significantly among habitats (Fig. 2c, Supplementary Fig. 8 and Supplementary Tables 9 and 10; the probability of 22 or more of the 35 tests achieving a *P* value of 0.05 is 1 × 10^−8^), sometimes with virus and host abundances correlating with different geochemical parameters (Supplementary Fig. 9 and Supplementary Table 11). However, no lineage revealed a progressive pattern with thaw across all three habitats. Instead, three broad patterns were observed: (1) an increase in virus/host abundance ratios with increasing thaw (for example, deltaproteobacterial viruses shifted from less abundant (palsa and bog) to more abundant (fen) than their hosts; Fig. 3a); (2) a decline in virus/host abundance ratios with increasing thaw (for example, viruses of the Solibacteres were less abundant in the bog and fen, relative to the palsa; Fig. 3b); and (3) invariable virus/host abundance ratios among habitats (for example, the Acidobacteriia and Nitrospirae and their respective viruses tended to be approximately equally abundant across the thaw gradient, with both virus and host abundances increasing from the palsa to the bog for the Acidobacteriia and from the palsa to the fen for the Nitrospirae; Fig. 3c,d). For both the Acidobacteriia and Nitrospirae, virus/host abundances were significantly correlated with pH and dissolved organic carbon (DOC) concentrations, with a particularly strong correlation with DOC for the Acidobacteriia, members of which are the primary degraders of large polysaccharides in the palsa and bog habitats^14^ (Fig. 3e and Supplementary Table 12). Although these analyses do not account for potential differences in virus and/or host activity, temporal offsets between virus and host peak abundances, or differences among palsas, bogs and fens beyond this single ecosystem, the results suggest that permafrost thaw may affect virus–host infection dynamics, with lineage-specific responses to biotic and/or abiotic parameters that distinguish habitats along the thaw gradient.

**Fig. 4:**
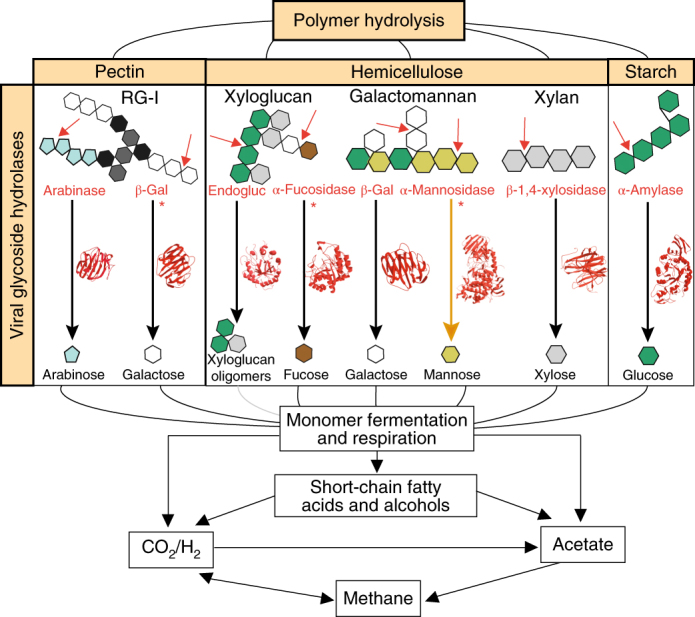
Potential viral contributions to carbon cycling in Stordalen Mire. Schematic overview of the carbon cycle, with light orange labels highlighting the potential viral contributions. Plant polymers that have the potential to be degraded by viral glycoside hydrolases are along the top, with examples of each below. Individual sugars are colour coded (see monomers beneath the black and orange arrows). The predicted enzymatic functions from computational protein models are listed in red, and example polymer cleavage points are indicated by the red arrows. Smaller molecules (monosaccharides and small oligosaccharides) cleaved from the polymers are shown at the ends of the black arrows, indicating the potential enzymatic conversion encoded by the viral glycoside hydrolase (the orange arrow indicates cleavage that was functionally confirmed by enzymatic assay), with the PHYRE2-modelled structure of each viral glycoside hydrolase in red. The asterisks indicate that multiple viral genes were recovered with the indicated predicted function. In those cases, the viral protein structure containing the most catalytic residues is shown (see Supplementary Table 14 for details). The grey line indicates a cleavage pathway of small oligosaccharides to monomers (non-viral). β-Gal, β-galactosidase; endogluc, endoglucanase; RG-I, rhamnogalacturonan I; black monomer, rhamnose; dark grey monomer, galacturonic acid.

To examine other means by which viruses might affect soil carbon cycling, we assessed whether Stordalen viruses contained auxiliary metabolic genes, as occurs in the oceans, impacting marine biogeochemistry (for example, via virus-encoded photosynthesis, central carbon metabolism, sulfur and nitrogen cycling genes^25^). Approximately one-fifth of the 59,416 predicted viral proteins (excluding those from predicted proviruses) could be functionally annotated and nearly all of those (96%) had predicted viral functions (Supplementary Table 13). The 4% remaining included 14 glycoside hydrolase genes, which were predicted via three-dimensional protein structural modelling to function in polymer hydrolysis, typical of bacteria and/or archaea, and were recovered on contigs conservatively validated as viral (346 additional glycoside hydrolase genes were identified but did not meet our conservative criteria). These 14 genes spanned 9 glycoside hydrolase families and had capacities for pectin, hemicellulose, starch and possibly cellulose cleavage (Fig. 4, Supplementary Table 14 and Supplementary Discussion). To test whether the predicted viral glycoside hydrolase genes could encode functionally active proteins, a viral glycoside hydrolase (from glycoside hydrolase family 5) was expressed and functionally assayed, confirming endomannanase activity with specific cleavage of β-1,4-linked mannose units in both glucomannan and galactomannan polymers (Supplementary Fig. 10). Together, these findings suggest that virus-encoded glycoside hydrolases may contribute to complex carbon degradation of plant-derived polymers to labile monosaccharides and small oligosaccharides (Fig. 4 and Supplementary Discussion), which fuel subsequent microbial carbon degradation, ultimately to CH_4_ and CO_2_. Consistent with previous evidence for horizontal gene transfer of lignin-degrading genes^30^, viruses could also potentially transfer glycoside hydrolases among hosts, conferring additional carbon degradation capacities. Future efforts will be necessary to confirm the activity and distribution of these viral glycoside hydrolases, along with the magnitude of viral contributions to terrestrial carbon cycling via glycoside hydrolases.

Finally, following previous efforts in marine systems^9^, we used partial least squares (PLS) regressions (see Supplementary Discussion) to investigate whether including viral abundances as explanatory variables could improve predictions of climate-relevant carbon chemistry measurements (response variables) beyond predictions using host abundances and/or abiotic factors alone. Specifically, we evaluated whether the abundances of viral populations that infected hosts with metabolisms of interest could help to predict three soil porewater carbon chemistry measurements: porewater CH_4_ concentrations, δ^13^C of porewater CH_4_ and DOC. Indeed, viral abundances often predicted porewater carbon chemistry, sometimes more significantly than host abundances (Supplementary Table 15, where tests of significance used a Bonferroni-corrected significance level of α = 0.00122). For example, δ^13^C of porewater CH_4_, an index of the methane production pathway and methane consumption used in ecosystem and climate models^6^, was not predicted by abiotic factors or methanogen and methanotroph host abundances, but was significantly predicted by methanogen and methanotroph viral abundances (*r* = 0.350, *P* = 3.6 × 10^−4^) and, especially, by the combination of methanogen and methanotroph hosts and their viruses (*r* = 0.424, *P* = 1.1 × 10^−^^5^). Decreases in methanogen viral abundances were associated with increases in δ^13^C of porewater CH_4_ (*r* = −0.390, *P* = 6.0 × 10^−^^5^), suggesting that declining viral predation of methanogens may contribute to the observed shifts in methane production from mostly hydrogenotrophic to more acetoclastic with thaw^6^.

Together, these results suggest that viral infections contribute to soil ecosystem functioning and that further interrogation of soil viral communities will yield a more comprehensive understanding of complex functional networks and ecosystem processes in soil. In addition, our observed correlations between viral abundances and a climate model-relevant biogeochemical index raise the question of whether a deeper understanding of soil viral ecology might provide a means to improve aspects of ecosystem and/or biogeochemical models.

## Methods

No statistical methods were used to predetermine sample size. Experiments were not randomized, and the co-authors were not blinded to allocation during experiments and assessments of the results.

### Sample collection

Bulk soil samples (*n* = 214) were collected as previously described^31^ from the Stordalen Mire long-term ecological research site in northern Sweden (68° 21′N, 19° 03′E, 359 m a.s.l. (above sea level)) during late spring, summer and early autumn months from 2010 to 2012 (Supplementary Table 1). Briefly, sets of triplicate soil cores were collected using a push corer from three site types along a permafrost thaw gradient: palsa (intact permafrost, frozen beneath the seasonally thawed near-surface active layer; samples were taken from the active layer, apart from the May 2012 frozen permafrost samples; see Supplementary Table 1), bog (partially thawed) and fen (fully thawed). Core diameters were as follows: 11-cm circular cores for all palsa samples and 10 × 10-cm square cores for all bog and fen samples^17^. Avoiding 1 cm around the edge of each core, cores were subsampled by depth, with each sample containing a 3-cm depth interval. Sampling depths ranged from 1 to 85 cm (average: 18 cm). Soil samples were placed in cryotubes, mixed to saturation with three volumes of LifeGuard solution (MoBio Laboratories) and stored at −80 °C until processing.

We also leveraged data from three small-size-fraction-enriched samples and three viral size-fraction samples that were collected from Stordalen Mire in 2014 for other purposes. Two of the small-size-fraction-enriched samples were from different depth ranges from a bog core and one was a fen sample (Supplementary Table 1). Briefly, each sample collected for small-size-fraction enrichment was homogenized in PBS and sequentially filtered through 3-μm, 1-μm, 0.8-μm, 0.4-μm, 0.2-μm and 0.05-μm filters. The bulk soil, 0.4-µm, 0.2-µm and 0.05-µm size fractions were sequenced from each of the three samples, and a co-assembly of those 12 libraries represents the small-size-fraction data set. For further details, see the ‘Small-size-fraction enrichment samples’ section below. For virome recovery, three soil samples were collected via push coring and stored either frozen (−20 °C) or chilled (4 °C) for the generation of seven total viromes (a combination of replicates and/or different storage conditions for the three samples; Supplementary Table 1). For further details, see the ‘Virome samples’ section below.

### DNA extraction, library construction and sequencing

Methods for DNA extraction from and sequencing of the 214 bulk soil metagenomes are as previously described^14,31^. Briefly (with a few exceptions^14^), 100 ng DNA per sample was used for TruSeq Nano (Illumina) library construction. The 2012 libraries yielded 100-bp paired-end reads from 1/12th of an Illumina HiSeq2000 lane, and the 2011 libraries yielded 150-bp paired-end reads from 1/24th of an Illumina NextSeq lane.

### Overview of how the 233 Stordalen Mire metagenomes were used

In total, 233 Stordalen Mire metagenomes were used in this study (214 bulk soil only, 12 small-size-fraction enriched and 7 viral size-fraction; Supplementary Table 1 and Supplementary Fig. 2). To generate a database of viral populations for maximal viral recovery from the bulk soil metagenomes, virus-affiliated sequences were mined from assemblies from: (1) 178 of the 214 bulk soil metagenomes (that is, all such metagenomes that were available at the time of this analysis, having been sequenced and assembled as of May 2015), (2) the 12 enriched small-size-fraction metagenomes, and (3) the 7 viral size-fraction metagenomes. All 214 bulk soil metagenomes were used for read mapping (see below), and through that mapping analysis, viral populations were detected in 201 metagenomes, resulting in the final data set of 201 bulk soil samples. All viral relative abundance data and subsequent ecological and statistical analyses were based on read mapping from those 201 bulk soil metagenomes to 1,907 recovered viral populations (the details for viral population recovery appear below). The small-size-fraction and virome data were used exclusively to promote viral recovery from the bulk soil metagenomes; no statistical analyses were performed on the size-fractionated samples.

### Sequence processing and assembly

A subset of the 214 bulk soil metagenomes (*n* = 178) was assembled using CLC bio’s de novo assembler (Qiagen) in three co-assemblies, according to habitat (one co-assembly each for palsa (*n* = 78), bog (*n* = 64) and fen (*n* = 36) samples; Supplementary Table 1).

### Recovering and annotating viral contigs

VirSorter was used to recover viral contigs, based on the identification of viral hallmark genes, enrichment in hypothetical proteins and other viral signatures, as previously described^32^. VirSorter was run separately on each of the five assemblies: the palsa, bog and fen bulk soil co-assemblies, the virome assembly and the small-size-fraction-enriched assembly (Supplementary Table 1). Only contigs from VirSorter categories 1 and 2 (and 4 and 5, the provirus equivalents of categories 1 and 2) were retained, based on a previous benchmarking study that found that, prior to manual curation, 100% of viruses in category 1 and 92% of viruses in category 2 were confirmed to be unambiguously viral^32^. Specifically, category 1 (and 4) viruses contain sequences similar to known viruses, and category 2 (and 5) viruses contain viral hallmark genes and/or are enriched for viral or non-Caudovirales genes and have at least one other virus-like metric (depletion of PFAM hits, hypothetical gene enrichment and/or depletions in coding-strand switching). For contigs with predicted proviruses, only predicted proviral regions were retained. After read mapping (see below), the data set of detected viral populations was manually curated to a final set of 1,907 viral contigs (viral populations) by ensuring that the VirSorter protein families (PFAM) annotation for each contig (Supplementary Table 13) contained genes that would be consistent with a viral genome, as described previously^18^. These methods probably resulted in the recovery of free viruses, proviruses and/or intracellular and potentially actively infecting viruses. As with any viral metagenomic study, some non-viral elements (regions of contigs or possibly full contigs) may have remained in the data set, even after manual curation.

### Viral population database compilation

To maximize our ability to recover viral populations from the 214 bulk soil metagenomes through read mapping, a fasta file combining viral contigs and genomes from different data sets was compiled (Supplementary Fig. 2). This initial viral database included the viral contigs recovered by VirSorter in this study from the bulk soil metagenomic assemblies (7,093 contigs; 3 phiX contigs were removed from the 7,096 initially recovered), the small-size-fraction-enriched assembly (401 contigs) and the virome assembly (53 contigs), along with the following publicly available data: 1,575 bacterial and archaeal viral genomes from the NCBI RefSeq database (v70, 26 May 2015)^20^, bacterial and archaeal viral genomes and genome fragments in Genbank but not in RefSeq as of July 2015 (1,147 contigs)^25^, and 12,498 viral contigs recovered and curated from publicly available bacterial and archaeal genomes^18^ (the Paez-Espino et al. data set^19^ was not published at the time of this mapping analysis but was incorporated into downstream analyses, see below). This viral database was dereplicated by clustering at 95% nucleic acid identity with Cd-Hit v4.6^33,34^. For clustering, all viral sequences recovered from Stordalen Mire were required to be ≥3 kb in length (smaller than the final data set of ≥10 kb, see below) and there was no length threshold for the publicly available sequences.

### Read mapping to the viral population database and viral operational taxonomic unit (OTU) table generation

Reads from the 214 bulk soil metagenomes were quality trimmed using Trimmomatic v0.36^35^ and then paired reads were mapped to the viral contig database with Bowtie2^36^, using default parameters. The output bam files were passed to BamM ‘filter’ v1.7.2 (http://ecogenomics.github.io/BamM/, accessed 15 December 2015) and reads that were aligned over ≥90% of their length at ≥95% nucleic acid identity were retained. A python script was used to further filter the bam files to ensure that all detected contigs had reads covering ≥70% of their length to minimize the potential for erroneous detection driven by small, potentially non-viral regions, and the coverage for a particular contig in a particular sample was converted to zero if this condition was not met. Finally, BamM ‘parse’ v1.7.2 was used to generate a coverage profile of viral contig abundances across samples, using the ‘tpmean’ coverage mode to account for anomalously low-coverage and high-coverage regions of each contig. The average per base-pair coverage for each contig in each sample was retained.

The final data set of 1,907 virus-affiliated sequences (populations) resulted from retaining only contigs that met the following criteria: ≥10 kb and/or circular, detected through read mapping in at least one of the 214 metagenomes, and PFAM annotation consistent with a viral origin. Thirteen metagenomes were removed from further analysis because no viral populations were detected according to these criteria, resulting in the final data set of 201 metagenomes across palsa (*n* = 72), bog (*n* = 65) and fen (*n* = 64) habitats.

The final ‘operational taxonomic unit (OTU) table’ (Supplementary Table 7) of viral abundances was pulled from the BamM mapping coverage output, normalized by the number of metagenomic reads in each sample. The normalization calculation was performed as follows: the average number of reads across all samples was determined, and, for a given sample, the total number of reads was divided by the all-samples average, and the reciprocal of that number was used as a multiplier to bring the total number of reads for each sample up to or down to the average (for example, all coverage values from a sample with twice as many reads as the average would have been multiplied by 0.5). For virus/host abundance ratios, this table was used directly, to best approximate actual abundances. For other statistical analyses, the normalized viral OTU table was subsequently square-root transformed.

### Protein clustering comparisons to publicly available viral sequences

To place the 1,907 Stordalen Mire viral populations in the context of known viruses, predicted proteins were clustered with predicted proteins from viral sequences in public databases. Specifically, the 1,907 viral populations were compared to: 2,010 bacterial and archaeal viral genomes from the NCBI RefSeq database (v75, June 2016)^20^, 2,040 viral contigs >10 kb from microbial genomes isolated from soil (pulled by habitat for this study from a larger data set^18^) and 3,112 viral contigs >10 kb from soil and soil-associated metagenomes (broadly defined soil-associated metagenomes were pulled by habitat for this study from a larger data set^19^, based on environmental labels in the original article as follows: ‘Terrestrial (soil)’, ‘Terrestrial (other)’, and ‘Host associated (plants)’). Proteins were then subjected to an all-versus-all BLASTp search with an E-value threshold of 10^−4^ and grouped into protein clusters, as previously described^25^. Based on the number of shared protein clusters between genomes and/or genome fragments (contigs), a similarity score for each pair was calculated as the negative logarithmic score by multiplying the hypergeometric similarity *P* value by the total number of pairwise comparisons, using vContact (https://bitbucket.org/MAVERICLab/vcontact, accessed 13 November 2016). The stringency of the similarity score was evaluated through 1,000 randomizations by permuting protein clusters or singletons (proteins without significant shared similarity to other protein sequences) within pairs of genomes and/or contigs having a significance score of ≤1 (a negative control)^37^. None of the genome and/or contig pairs in this negative control produced significant scores of >1, indicating an appropriately defined similarity score threshold^38^. Subsequently, pairs of genomes and/or contigs with a similarity score of >1 were clustered into viral clusters with the Markov clustering algorithm with an inflation of 2, as previously described^25^. The resulting network was visualized with Cytoscape software (version 3.4.0, http://cytoscape.org/), using an edge-weighted spring embedded model, which places the genomes and/or contigs that share more protein clusters in closer proximity in the display. Note that this analysis (which clusters viral contigs at approximately the genus level based on shared protein content^22^) was used exclusively for taxonomic assignments and comparisons to public databases; for all other analyses, viral populations were clustered at approximately the species level, based on whole-genome (or whole-contig) nucleotide identity, as described in the preceding section on read mapping.

### Viral taxonomic assignments

Reference sequences from the 2,010 RefSeq genomes that co-clustered with the Stordalen Mire viral populations through the protein clustering analyses described above were leveraged for predicting taxonomy. Only reference sequences with near-complete viral lineage assignments were used for taxonomic predictions. Briefly, based on the percentage of reference sequences comprising a given viral cluster at each taxonomic level, each viral cluster was given a high-, medium- or low-confidence score for its taxonomic assignment (≥50%, ≥30% and ≥10% cut-offs, respectively). In addition, a last common ancestor approach was applied to all reference sequence-containing viral clusters in which RefSeq genomes of differing taxonomy were clustered. In these cases, the highest taxonomic level in common for the majority of reference sequences was retained.

### Viral population abundances in metatranscriptomes

Metatranscriptomic data generation was described previously^14^. Briefly, most metatranscriptomic libraries were run on either a HiSeq (Illumina) or MiSeq (Illumina) to assess library quality before deeper NextSeq (Illumina) sequencing. Before metatranscriptomic read mapping to viral population sequences, part of the TranscriptM pipeline (https://github.com/elfrouin/transcriptM) was used to process metatranscriptomic reads, as follows: sequencing read files from the same metatranscriptomic libraries were concatenated across sequencing runs. Raw reads were trimmed via Trimmomatic^35^, using quality scores determined via FastQC (http://www.bioinformatics.babraham.ac.uk/projects/fastqc/). PhiX contamination was removed by discarding reads that aligned to the PhiX genome via BamM mapping. SortMeRNA^39^ was used to remove non-coding RNA sequences, including transfer RNA, transfer-messenger RNA, 5S, 16S, 18S, 23S and 28S rRNA sequences. The remaining total mRNA sequences were used for mapping to the 1,907 viral population contig sequences to infer the composition of the active viral community in 21 bulk soil metatranscriptomes collected from a subset of the same samples from which the 201 metagenomic samples were generated (Supplementary Table 1 and Supplementary Fig. 2). Mapping parameters were the same as for metagenomic read mapping, that is, ≥95% nucleotide identity, ≥90% of each read mapped and the ‘tpmean’ output from BamM v1.7.2 (http://ecogenomics.github.io/BamM/). Dirseq v0.0.2 (https://github.com/wwood/dirseq) with parameter -ignore-directions was used to determine the average coverage of each gene, using both the filtered bam files and PROKKA annotation.gff files as input. A positive average coverage value (indicating read mapping to a gene) for at least one gene for every 10 kb of viral genomic sequence was required for a viral population to be considered detected in a metatranscriptome (for example, for a 20-kb viral sequence, metatranscriptomic reads would need to map to at least two genes for detection). Supplementary Table 2 includes the final coverage table used in the metatranscriptomic data analyses, derived from average coverage values for all genes in each viral population, provided that the population met detection limits (if limits were not met, the coverage value for a particular population in a particular metatranscriptome was converted to zero). Although these methods are meant to provide an estimate of active viral community composition, there is no standardized method or biological precedent for assessing viral activity via detection in a bulk soil metatranscriptome, so the extent to which these methods yield an accurate estimate of viral activity is unknown.

### Microbial (‘host’) population genomes and relative abundances

Bacterial and archaeal population genomes (*n* = 1,529) were recovered from the same 214 bulk soil metagenomes through metagenomic assembly and differential coverage binning, as previously described^14^. For comparisons to viral abundances, bacterial and archaeal (‘host’) abundance profiles were generated from the 201 bulk soil metagenomes in which viral populations were detected, using a mapping approach similar to that used for viral abundance estimates. The 1,529 bacterial and archaeal population genomes were first dereplicated at a 95% average amino acid identity (AAI) to minimize multi-mapping (that is, reads mapping to more than one genome). The AAI was calculated between genomes using the CompareM (v.0.0.17) AAI workflow (‘comparem aai_wf’, https://github.com/dparks1134/CompareM). Genomes with >95% AAI were grouped together, and the highest quality genome in each group was chosen as the representative, where quality was estimated by CheckM^40^ as ‘completeness - 4 × contamination’. To calculate the relative abundance of each population, reads from each of the 214 bulk soil metagenomes were mapped to the set of 630 dereplicated genomes using BamM ‘make’ (http://ecogenomics.github.io/BamM/). Low-quality read mappings were removed with BamM v1.7.3 ‘filter’ (minimum nucleotide identity of 95%, minimum aligned length of 75% of each read), and the coverage of each contig was calculated with BamM ‘parse’ using the ‘tpmean’ mode to calculate the coverage as the mean of the number of reads aligned to each position, after removing the highest 10% and the lowest 10% coverage regions. The coverage of each population genome was calculated as the average of all of its binned contig coverages, weighting each contig by its length in base pairs. The final host OTU table (Supplementary Table 8) of bacterial and archaeal abundances was pulled from the BamM mapping coverage output, normalized by the number of metagenomic reads in each sample, calculated as described above for the viral OTU table. Only average coverage values of ≥0.25× were retained; lower values were converted to zero. For virus–host abundance analyses, this table was used directly, and for Mantel correlations, this normalized OTU table was square-root transformed.

### Virus–host linkage analyses

Where possible, the 1,907 Stordalen Mire viral populations were putatively linked to host population genomes^14^ in silico, using similar methodology to previous work^25,29^, with a few enhancements. Broadly, these linkages were based on: (1) sequence similarity between spacers in microbial clustered regularly interspaced short palindromic repeat (CRISPR) regions and in the viral genomes (that is, viral protospacers), as previously described^19,25,29,41^, with the addition of a priority for linkages in which a protospacer adjacent motif^42^ was recovered in the viral contig, (2) similarities in tetranucleotide frequency patterns^25^, and (3) shared genomic content, similar to previous work^19,25^. To recover CRISPR spacer and repeat elements from metagenomic reads, crass v0.3.6 was used with default parameters, running on each of the 214 bulk soil metagenomes separately^29,43^. BLASTn was used to compare spacer sequences to the viral contigs, with matches retained if they contained ≤1 mismatch and had an E-value of ≤10^−5^. For any spacer with a match in a viral genome, the repeat sequence from the same assembled CRISPR region was compared to all bacterial and archaeal population genomes via BLASTn (E-value threshold of 10^−10^ and 100% nucleotide identity) to link that CRISPR region (and, therefore, any viruses matching spacers in that CRISPR region) to a host. Tetranucleotide frequency patterns^25,44^ were assessed independently for viral populations and hosts, and for each viral population, the host genome with the most similar tetranucleotide frequency pattern was identified as a putative host, with a threshold of 10^−3^ on the distance between the host and the viral sequence^25^. Finally, BLASTn was used to link viral populations to hosts, based on shared genomic regions, which could indicate either shared genes between viruses and hosts (for example, auxiliary metabolic genes and/or tRNAs^19,25^) and/or could be indicative of proviruses. A bit score threshold of 50, an E-value threshold of 10^−3^ and a ≥70% average nucleotide identity were required, and only hits ≥2,500 bp were considered, as these have been previously shown to yield the most confident host predictions^45^. Among these hits, a BLASTn hit that covered ≥90% of a contig in a microbial genome bin was interpreted as a viral contig that was ‘co-binned’ with a microbial genome. These were considered less-confident host predictions than proviruses (defined as matching ≤90% of a contig in a microbial genome bin). All predictions from all metrics are available in Supplementary Table 5.

For virus–host abundance estimates, a single predicted host was chosen for each viral population. We realize that this does not account for the possibility of broad host ranges, as reported in Supplementary Table 5, but it was necessary for the analysis. The following priority order of linkage metrics (the most robust to the least robust) was used to identify a single predicted host for each viral population: (1) CRISPR linkage with a protospacer adjacent motif, (2) CRISPR linkage without a protospacer adjacent motif, (3) BLASTn linkage to ≤90% of a contig in a microbial genome bin (putative provirus), (4) BLASTn linkage to ≥90% of a contig in a microbial genome bin (a putative virus co-binned with the host), and (5) the best-matched tetranucleotide frequency patterns. If multiple hosts were predicted in the best of those five categories, then the last universal common ancestor was chosen as the host, based on the lowest (most highly resolved) shared taxonomic level in the predicted host taxonomies. Host taxonomy information for all analyses was based on available reference database sequences as of August 2016 and has subsequently been updated^14^ (Supplementary Table 8).

### Virus–host abundance estimates

From the normalized OTU tables of microbial population genome abundance and viral abundance, respectively, microbial host abundances were summed at the class level (or at the phylum level if the class was not informative or was not well populated for a particular lineage; Supplementary Table 8), and viral population abundances were summed at the same taxonomic level for their predicted hosts. These summed host and viral abundances were used to calculate ‘host lineage-specific’ virus/host abundance ratios and for comparisons of the abundances of both viruses and hosts across samples. Similarly, for host metabolism-specific analyses (Supplementary Table 15), the abundances of all hosts predicted to encode particular metabolic processes, based on the presence of relevant biogeochemical pathways in the microbial population genome bins (Supplementary Tables 6 and 8), were summed, and the abundances of all viruses predicted to infect those hosts were also summed.

### Auxiliary metabolic gene analysis, recovery of viral glycoside hydrolases and experimental characterization of a viral GH5

Of the 1,907 viral populations, 1,743 VirSorter-predicted non-proviral contigs (that is, VirSorter categories 1 and 2) were considered for these analyses, to minimize potential host genome contamination (for example, from imperfectly called proviral ends). All genes underwent a re-annotation, as described previously^46,47^. Briefly, open reading frames were predicted using MetaProdigal^48^, and predicted protein sequences were compared to the InterProScan^49^ database via USEARCH^50^, with single and reverse best-hit matches of >60 bits retained. As previously described^46^, glycoside hydrolase genes were manually pulled using PFAM identification numbers from InterProScan associated with carbohydrate-active proteins. In total, 360 genes were annotated as glycoside hydrolases, 293 of which were identified as chitinase, lysozyme or putative cell wall-binding genes that have previously been shown to occur in viral genomes with predicted viral functions^51,52^. The remaining 65 glycoside hydrolases were predicted to degrade cellulose, hemicellulose, starches or pectin, and these were manually curated to a conservative set of 24 glycoside hydrolases that had unambiguous virus-like genomic contexts (Supplementary Table 14). For ‘high-confidence’ viral genomic contexts, common viral genes, for example, viral structural genes, terminases or integrases, were required to be found in genomic regions both upstream and downstream of the glycoside hydrolase, and for both high-confidence and medium-confidence viral genomic contexts, no other microbial metabolic genes could be found on any part of the contig, as those could indicate a possible microbial origin for the glycoside hydrolase, for example, resulting from mispackaging of the viral genome or incorrectly called proviral ends by VirSorter.

Protein sequences from the 24 glycoside hydrolases were structurally modelled using PHYRE2 in expert batch submission mode (http://www.sbg.bio.ic.ac.uk/phyre2/html/page.cgi?id=index) to confirm and further resolve functional predictions. Of these, 14 had 100% confidence scores to bacterial or archaeal glycoside hydrolases and were further investigated for catalytic residues in active sites (the other 10 had potentially virus-like functional predictions or no match in the PHYRE2 database). Catalytic residues were compared with reference sequences, using the Catalytic Site Atlas^53^ when available, otherwise crystal structures were manually identified using references from the Protein Data Bank (Supplementary Table 14).

The amino acid sequence encoded by a predicted glycoside hydrolase family 5 (*GH5*) gene recovered from the bog assembly (NCBI-abbreviated contig ID: Bog_ctg_46288_cat2) was optimized for *Escherichia coli*, synthesized and cloned into the pET151/D-TOPO plasmid, by Invitrogen GeneArt. The plasmid was transformed into *E.* *coli* One Shot BL21 Star cells (Thermo Fischer Scientific) and an overnight pre-culture was inoculated to 1% in 50 ml Lysogeny Broth with 100 mM ampicillin, incubated at 37 °C and shaken at 180 r.p.m. until reaching an optical density at 600 nm of 0.6. Cells were chilled to 18 °C in a water bath, and expression was induced by adding isopropyl-β-D-thiogalactopyranoside to a final concentration of 0.25 mM. The culture was incubated at 18 °C, shaken at 180 r.p.m. for 6 h and cells were harvested by centrifugation (4,500*g* for 20 min). Cells were washed once in 50 mM Tris-HCl, 0.2 M NaCl, 5 mM imidazole, pH 8.0, before resuspension in 400 µl of 20 mM sodium phosphate, 300 mM sodium chloride (PBS) with 10 mM imidazole, pH 7.4, and sonicated for 3 × 15 s (20% amplitude, 1 s + 1 s pulses) using a Vibracell sonicator (Sonics). Cell lysate was obtained by centrifugation (14,000*g* for 15 min) and purified using HisPur Ni-NTA Spin Columns (Thermo Fisher Scientific) according to the manufacturer’s instructions. Purification fractions were examined by SDS–PAGE, and protein concentration was calculated from A280 using the estimated extinction coefficient of the expressed protein. Enzymatic assays were performed in a 96-well plate and contained 20 mM phosphate buffer pH 4.0 and 0.5% glucomannan (konjac, low viscosity; Megazyme), galactomannan (carob; Megazyme), β-glucan (barley, medium viscosity; Megazyme), carboxymethylcellulose (low viscosity; Sigma), xyloglucan (tamarind; Megazyme), arabinoxylan (wheat; Megazyme) or carboxymethyl-pachyman (Megazyme). Reactions were pre-heated (40 °C for 10 min) in a Thermomixer C incubator with a heated lid (Eppendorf), before the addition of the enzyme to 0.5 µM (final reaction volume: 100 µl) for further incubation (60 min). The reactions were stopped by the addition of an equal volume of 1% DNS reagent (10 g l^–1^ 3,5-dinitrosalicylic acid, 300 g l^–1^ potassium sodium tartrate, 10 g l^–1^ NaOH^54^), and the sealed plate was heated to develop colour (95 °C for 20 min). Heat-treated samples (150 µl) were transferred to a new plate, and A540 was measured in a Multiscan FC Microplate Photometer. Released reducing ends were quantified against a standard curve of glucose. For product analyses from mannan substrates, konjac glucomannan, carob galactomannan and guar galactomannan were degraded as described above with an extended incubation time of 18 h. Reactions were stopped by the addition of NaOH to a final concentration of 0.1 M. Products were analysed by High-pH anion-exchange chromatography–pulsed amperometric detection on a Dionex ICS-5000 system with a CarboPac PA1 column at a flow rate of 0.25 ml min^−1^. Oligosaccharides were eluted in a multistep linear gradient, as follows: 0–9 min, 0.1 M NaOH; 9–35 min, 0.1 M NaOH with a linear sodium acetate (NaOAc) gradient 0–0.3 M; 35–40 min, 0.1 M NaOH, 0.3 M NaOAc; 40–50 min, 0.1 M NaOH.

### Environmental, geochemical and nutrient measurements

Environmental, geochemical and nutrient measurements include depth, temperature, moisture content, pH, elemental and nutrient compositions and isotopic data (Supplementary Table 1), and some of the approaches for these measurements have been described previously^2,6,31,55,56^.

#### Temperature

Temperatures in 2011 were measured in the ambient air and at 3 cm and 13 cm below either the peat surface (if the water table depth is ≤0) or the water table surface (if the water table depth is >0), using an EBRO Thermometer TFX 392L. Temperatures of the air and peat at each sample depth in 2012 were measured with a 60-cm stainless steel probe connected to a HH507RA Multilogger Thermometer (Omega Engineering).

#### Pore water and pore gas sampling

Pore water (at water-saturated depths; Supplementary Table 1) and pore gas (at water-unsaturated depths) were collected by suction with a 60-ml plastic syringe connected to a 1/4-inch stainless steel tube with holes drilled along the bottom 3 cm. Pore gas samples (>30 ml for each sample) were injected directly into 30-ml evacuated borosilicate glass vials sealed with butyl rubber septa. Pore water for the measurement of CH_4_ and dissolved inorganic carbon concentrations and δ^13^C values was filtered through Whatman GF/D glass microfibre filters (2.7-μm particle retention) and injected into 20-ml or 30-ml evacuated vials, similar to those used for pore gas, until the vials were 50–70% full. Duplicate vials were filled for each depth. Pore water for the measurement of pH and nutrient concentrations (DOC, total nitrogen, acetate, sulfate, nitrate and ammonia) was filtered through 0.7-μm Whatman GF/F glass microfibre filters into 120-ml brown borosilicate bottles. All pore water was frozen within 8 h of collection and kept frozen until analysis.

#### pH

Pore water pH was measured in the field with an Oakton Waterproof pHTestr 10 (Eutech Instruments). For unsaturated soils with insufficient pore water for direct pH measurement (for example, palsa and surface bog samples), a small amount (~1 g) of wet soil was vigorously mixed with 1–2 ml deionized water with a stainless steel spatula to create a slurry. The pH of the slurry was then measured with the same pH meter used for the field measurements.

#### Solid-phase soil properties

Soil was dried at 60 °C and the soil moisture content (%) was determined gravimetrically based on the difference between wet and dry weight. The dried soil was then ground to a fine powder and analysed for %C, %N and C/N ratios (by weight) using combustion elemental analysis/isotope ratio mass spectrometry^2^. Isotopic compositions (δ^13^C and δ^15^N) were measured at the same time as the total carbon and nitrogen contents. δ^13^C values were defined relative to the Vienna PeeDee Belemnite (VPDB) standard, where δ^13^C = [*R*_sample_/*R*_standard_ − 1] × 1,000, and *R*_sample_ and *R*_standard_ are the ^13^C/^12^C ratios in the sample and the standard. A similar definition was used for δ^15^N values, with N_2_ (air) used as the standard.

#### Gas concentrations and δ^13^C values

Our methods for sample preparation and analysis of gas concentrations and δ^13^C values have been described previously^2,6,31,56^. Prior to measurement, pore water samples were thawed, acidified with 0.5 ml degassed 21% phosphoric acid (H_3_PO_4_) (excess) and brought to atmospheric pressure with helium. Pore gas samples and pore water sample headspaces were then measured for CH_4_ and CO_2_ concentrations (% volume) and δ^13^C values (relative to the VPDB standard) by gas chromatography/isotope ratio mass spectrometry. For pore water, headspace gas concentrations were converted into dissolved CH_4_ and DIC concentrations (mM), as previously described^56^.

#### DOC and total dissolved nitrogen

Concentrations of DOC and total dissolved nitrogen were measured in triplicate via high-temperature catalytic oxidation on a Shimadzu Total Organic Carbon analyzer with a non-dispersive infrared detector^55^.

#### Acetate and phosphate

Acetate and phosphate concentrations were analysed by ion chromatography on a Dionex DX600, fitted with a 4-mm AS-11HC column with suppression, and with a potassium hydroxide eluent generator. These measurements were performed at the University of Massachusetts, Lowell (Lowell, MA, USA).

#### Sulfate and nitrate

Concentrations of sulfate (all samples) and nitrate (samples collected in July 2011 and later) were measured by ion chromatography on a Dionex ICS-1100 with a 4-mm IonPac AS22 column, an eluent of 4.5 mM carbonate/1.4 mM bicarbonate and a flow rate of 1.2 ml min^−1^. Nitrate concentrations for the June 2011 samples were measured by reduction to gaseous NO in an acidic VnCl_3_ solution, followed by chemiluminescence detection with a Thermo model 42i NO_x_ analyzer (Thermo Scientific)^57^.

#### Ammonium

Ammonium concentrations were measured by reaction with hypochlorite and salicylate followed by colourimetric absorbance measurements at 640 nm^58^.

### PLS regression analysis

We used a PLS regression analysis, implemented in the R programming language via the package PLS and PLSR function^9,59,60^ to predict measured geochemical variables (response variables) from biotic and abiotic variables (explanatory variables). PLS models a causal relationship between an explanatory variable (or variables; in this case, the abundances of subgroups of viruses and/or hosts and/or abiotic factors) and the response variable being predicted. We note that, in this analysis, Stordalen Mire is treated as a single system with varying biogeochemical, microbial and viral indices that can be related by statistical analysis as a means for characterizing their interrelationships within this site only. This PLS analysis assumes that different samples are statistically independent, and we believe that this assumption is met here because the distance between samples (several metres at minimum, extending to tens of metres; see Supplementary Fig. 1) is large compared to the typical scale of spatial variation in terrestrial microbial communities (for example, see Franklin and Mills, 2007 and the references therein)^61^.

We predicted three measured variables (response variables) related to carbon cycling (porewater CH_4_ concentrations, DOC concentrations and δ^13^C of porewater CH_4_), which were previously shown to vary across the thaw gradient^2,6^. For each response variable, we first selected the most relevant subgroup(s) of viruses to use as predictors (explanatory variables) from a set of metabolism-specific and lineage-specific subgroups (specifically, viruses of: methanogens, methanotrophs and the four host lineages highlighted in Fig. 3 for their virus–host dynamics across the thaw gradient, that is, Deltaproteobacteria, Solibacteres, Acidobacteriia and Nitrospirae, each of which has multiple biochemical pathways for complex carbon degradation but not methanogenesis or methanotrophy; Supplementary Table 6). If no combination of selected viral groups significantly predicted a given response variable, no further predictors were considered for that response variable. If at least one viral group was a significant predictor of a given response variable, then different combinations of viruses, hosts and abiotic factors were considered to evaluate the relative viral contributions to the prediction of that response variable (Supplementary Table 15). When abiotic factors were considered as explanatory variables (predictors), all ten of the following variables from Supplementary Table 1 were included: depth (cm), soil temperature (°C), pH, soil moisture content (%), nitrogen content (dry weight %), δ^15^N and the concentrations of nitrogen, sulfate, nitrate and ammonia (mM). Those variables were selected, based on the following requirements: measurements were sample specific (as opposed to, for example, common to all samples within a core), a measurement was taken for at least 30% of the samples and all measurements of carbon-containing compounds were excluded to minimize the potential for confounding with the variable being predicted. Although we have selected all possible geochemical variables for which we have a reasonably complete data set, along with all known methanogen and methanotroph host lineages and their predicted viruses, we acknowledge that selecting specific variables could lead to biases in the model and, therefore, in its interpretation. The PLS analysis yielded Pearson’s product moment correlations between the abundances of specific viral and microbial groups and measured carbon chemistry variables, allowing for a quantification of the added value of viral abundances in predicting carbon chemistry, relative to predictions from the abundances of microorganisms and abiotic factors when virus information was not included.

### Small-size-fraction enrichment samples

#### Sample preparation and DNA extraction

Three fresh samples were shipped from Stordalen Mire in July 2014 and kept at 4 °C during transport and storage. These included two samples from different depth ranges from a bog core (20–24 cm and 40–44 cm, respectively) and a fen sample (20–24-cm depth). The three samples were kept anaerobic at 4 °C during the filtration process. The following protocol was used for each sample: 7 g of soil were homogenized using a mortar and pestle and 1× PBS was added to a final slurry volume of 15 ml. The sample was then sonicated, using a sonication bath, for 3.5 min to dissociate cells from organic material, centrifuged at 1,000*g* for 2 min and the supernatant was filtered through a 100-µm sieve. The organic matter pellet from the centrifugation was strained using a 100-µm sieve, washed with PBS and strained again. Nycodenz (2 ml) was added to two 15-ml falcon tubes, and 6 ml of slurry was carefully placed above the Nycodenz cushion of each tube. These tubes were centrifuged at 3,270*g* for 75 min at 4 °C to separate the cells from the organic matter homogenate. The aqueous phase, cell layer, and half of the Nycodenz cushion were combined from both tubes and briefly vortexed. The slurry was stored overnight until filtration the next day. After brief vortexing, the samples were put through a 3-µm filter, which was then flushed with 2 ml PBS. Subsequently, two 1-µm filters were required due to filter clogging, and 1 ml PBS was pushed through each filter following sample filtration. The post-1-µm filtrate was put through one 0.8-µm filter, followed by 1 ml PBS. Owing to 0.4-µm filter clogging, each sample was put through three 0.4-µm filters, followed by 1 ml PBS. The total sample volume was subsequently put through a 0.2-µm filter and retained on a 0.05-µm filter. Filters were cut and placed into MoBio PowerSoil tubes before the addition of the lysis buffer and set on a shaker at 100 r.p.m. for 1 h to help to disperse the cells from the filter. The MoBio PowerSoil protocol was followed according to the manufacturer’s instructions for the remainder of the DNA extraction. DNA concentrations were calculated using the Qubit HS dsDNA kit (Life Technologies), and the bulk samples and 0.4-µm, 0.2-µm and 0.05-µm fractions of each sample were selected for library preparation using the Nextera XT kit (Illumina). Libraries were successful for each of the fractions (3 samples × 4 fractions = 12 libraries), and each library was sequenced as 2 × 150 bp paired-end reads on 1/13th of a NextSeq (Illumina) lane.

#### Computational workflow

Initial quality control of the 12 sequence data files was performed using SeqPrep (https://github.com/jstjohn/SeqPrep) for adaptor trimming and merging of the paired-end reads, and Nesoni (https://github.com/Victorian-Bioinformatics-Consortium/nesoni) was used for trimming poor-quality bases. De novo assemblies of the quality-processed sequencing data were constructed using CLC Genomics Workbench (CLC bio). A combination of all 12 metagenomes yielded the best assembly (the ‘ultrasmall’ assembly in Supplementary Table 1), and the contigs from this assembly were submitted to VirSorter to mine for viruses, as described above.

### Virome samples

#### Sample collection and storage

Three soil samples were collected in July 2014 via push coring and stored either frozen (−20 °C) or chilled (4 °C) for the generation of seven viromes (Supplementary Table 1). Specifically, one palsa, one bog and one fen sample were collected. Two viromes were generated from the palsa sample (one was stored frozen and one was chilled), three viromes were generated from the bog sample (two were from replicate fractions stored frozen and one was from a fraction stored chilled) and two viromes were generated from the fen sample (both were replicate fractions stored chilled).

#### DNA extraction and sequencing

After viral purification^17^, viral DNA was extracted from each of the seven subsamples, using Wizard columns and resin (A7181 and A7211, Promega), cleaned with AMPure beads (A63881, Beckman Coulter) and concentrated with Zymo DNA Clean and Concentrate-5 (D4004), all according to the manufacturers’ instructions. The Nextera XT DNA Library Preparation Kit (Illumina) was used for library construction, and the seven viromes were sequenced using an Illumina MiSeq (V3 600 cycle, six samples per run) at the University of Arizona Genetics Core facility (Tucson, AZ, USA).

#### Bioinformatic processing

Reads were quality-trimmed using Trimmomatic^35^ (adapters were removed and reads were trimmed from ends, starting from regions with an average per-base quality score below 20 on 4-nucleotide sliding windows; remaining reads shorter than 50 bp were discarded). IDBA_UD^62^ was used for a single co-assembly of all seven viromes with default parameters. Contigs were processed with VirSorter^32^ in virome decontamination mode and were also compared to putative laboratory contaminants (that is, phages cultivated in the M.B.S. laboratory: enterobacteria phage PhiX17, Alpha3, M13, *Cellulophaga baltica* phages and *Pseudoalteromonas* phages) via BLASTn. Contigs with >95% average nucleotide identity to these genomes were removed. Fifty-three contigs ≥10 kb in length were retained and used as part of the mapping database for viral population detection in the 214 Stordalen Mire bulk soil metagenomes (Supplementary Fig. 2).

### Statistics

For statistical comparisons of viral community composition (the viral OTU table) to environmental and geochemical parameters and to host community composition (the host OTU table), analyses were performed using PRIMER v6^63,64^. A Bray–Curtis dissimilarity matrix was generated from the square-root transformed viral OTU table for sample comparisons. Permutational multivariate analysis of variance (PERMANOVA; 99,999 permutations) was used to test for significant differences in viral community composition among the three thaw habitats. Mantel tests with Spearman’s rank correlations (999 permutations) were used to compare viral community composition to continuous and/or multidimensional variables, including environmental and geochemical data (Euclidean distance matrices) and host community composition (Bray–Curtis dissimilarity matrix constructed from the square-root transformed host OTU table).

Pearson correlations were performed using SigmaPlot 11.0 (Systat Software) to assess the relationships between the abundances of viruses, their hosts and environmental variables in all samples, as well as in individual habitats (palsa, bog and fen) (Figs. 2c and 3e, Supplementary Fig. 9 and Supplementary Table 11).

Relationships between viral population and host population abundances within and across habitats were investigated using linear regression analyses in R v3.3.1^65^. The linear regression models for each lineage in each habitat were then compared using two-way analysis of variance (ANOVA) to test whether the interaction term in the linear regression models (that is, designating samples as palsa, bog or fen) was significantly different from not using an interaction term (that is, all samples included in the regression model) for a given host lineage (Supplementary Table 9).

As some of our analyses included statistical tests with multiple comparisons, we adjusted estimates of statistical significance to account for this in two ways. First, for tests of multiple specific hypotheses (the PLS regressions, in which the goal was to identify specific predictive relationships with confidence), we used the Bonferroni correction to adjust our target significance by the number of regressions tested (that is, for *n* regressions, significance was achieved when *P* *<* α/*n*). Thus, the criteria for a regression to achieve significance at the α = 0.05 level in the context of the 41 PLS regressions tested in Supplementary Table 15 was *P* *<* 0.05/41 = 0.00122. Second, for tests that were used to generally quantify the pervasiveness of correlations between ecological indices (that is, viral and host lineage abundances) and between those indices and biogeochemical indicators, we used a binomial test to compare the number of correlations observed to be significant at α = 0.05 to the null distribution of the number of correlations expected to test as significant under the null hypothesis of no correlation (that is, the number of expected false positives), given the number (*n*) of tests conducted (for example, when α = 0.05, *n* × 0.05 correlations are expected to appear significant even when the null hypothesis is true). Because the observed number of correlations with *P* < 0.05 substantially exceeded the minimum number of ‘successes’ expected under the binomial distribution in all cases, we considered the correlations pervasive enough to be significant.

### Reporting Summary

Further information on experimental design is available in the Nature Research Reporting Summary linked to this article.

### Code availability

The mapping pipeline is freely available on CyVerse^66^ as ‘Read2RefMapper-1.1.0’.

### Data availability

The sequencing data described in this article, including metagenomic and metatranscriptomic data also described in Woodcroft et al.^14^, are submitted under NCBI BioProject accession number PRJNA386568. Within that BioProject, the 1,907 viral population contig sequences have been deposited at DDBJ/ENA/GenBank under the accession number QGNH00000000. The version described in this paper is version QGNH01000000. The Supplementary Information includes 15 supplementary data sets (Supplementary Tables 1–15). Other data that support the findings of this study are available from the corresponding author upon request.

## Supplementary information

Supplementary Information Supplementary Discussion, Supplementary Figures 1–10

Reporting Summary

Supplementary Dataset 1 Supplementary Tables 1–15
