# Supplementary material for: Host-linked soil viral ecology along a permafrost thaw gradient
Source: Nat Microbiol. 2018 Jul 16;3(8):870–80. doi: 10.1038/s41564-018-0190-y (PMC6786970; doi:10.1038/s41564-018-0190-y)
Supplement: Supplementary file 1 — Supplementary Discussion, Supplementary Figures 1–10 [file 41564_2018_190_MOESM1_ESM.pdf]

nature microbiology

SUPPLEMENTARY INFORMATION

https://doi.org/10.1038/s41564-018-0190-y

In the format provided by the authors and unedited.

Host-linked soil viral ecology along a permafrost thaw gradient

Joanne B. Emerson[1,9], Simon Roux[1,10], Jennifer R. Brum[1,11], Benjamin Bolduc[1], Ben J. Woodcroft[2], Ho Bin Jang[1], Caitlin M. Singleton[2], Lindsey M. Solden[1], Adrian E. Naas[3], Joel A. Boyd[2], Suzanne B. Hodgkins[4], Rachel M. Wilson[4], Gareth Trubl[1], Changsheng Li[5,12], Steve Frolking[5], Phillip B. Pope[3], Kelly C. Wrighton[1], Patrick M. Crill[6], Jeffrey P. Chanton[4], Scott R. Saleska[7], Gene W. Tyson[2], Virginia I. Rich[1] and Matthew B. Sullivan[1,8]*

[1]Department of Microbiology, The Ohio State University, Columbus, OH, USA. [2]Australian Centre for Ecogenomics, School of Chemistry and Molecular Biosciences, University of Queensland, Brisbane, Brisbane, Queensland, Australia. [3]Faculty of Chemistry, Biotechnology and Food Science, Norwegian University of Life Sciences, Ås, Norway. [4]Department of Earth, Ocean, and Atmospheric Science, Florida State University, Tallahassee, FL, USA. [5]Earth Systems Research Center, Institute for the Study of Earth, Oceans and Space, University of New Hampshire, Durham, NH, USA. [6]Department of Geological Sciences, Stockholm University, Stockholm, Sweden. [7]Department of Ecology and Evolutionary Biology, University of Arizona, Tucson, AZ, USA. [8]Department of Civil, Environmental and Geodetic Engineering, The Ohio State University, Columbus, OH, USA. [9]Present address: Department of Plant Pathology, University of California, Davis, Davis, CA, USA. [10]Present address: United States Department of Energy Joint Genome Institute, Lawrence Berkeley National Laboratory, Walnut Creek, CA, USA. [11]Present address: Louisiana State University, Baton Rouge, LA, USA. [12]Deceased.
*e-mail: mbsulli@gmail.com

Supplementary Information for
Host-linked soil viral ecology along a permafrost thaw gradient

Joanne B. Emerson, Simon Roux, Jennifer R. Brum, Benjamin Bolduc, Ben J. Woodcroft, Ho Bin Jang, Caitlin M. Singleton, Lindsey M. Solden, Adrian E. Naas, Joel A. Boyd, Suzanne B. Hodgkins, Rachel M. Wilson, Gareth Trubl, Changsheng Li, Steve Frolking, Phillip B. Pope, Kelly C. Wrighton, Patrick M. Crill, Jeffrey P. Chanton, Scott R. Saleska, Gene W. Tyson, Virginia I. Rich & Matthew B. Sullivan

Supplementary Information includes Supplementary Discussion, 10 Supplementary Figures, and 15 Supplementary Tables (separate file).

Supplementary Discussion

Viral glycoside hydrolases

The 14 identified "bacteria- or archaea-like" viral glycoside hydrolases (GHs) had 100% Phyre2 confidence scores to bacterial and archaeal GHs in 9 CAZy (Carbohydrate-Active Enzyme) families[1], including: GH2 (β-galactosidases, β-glucuronidases, β-mannosidases), GH5 (endoglucanases, endomannanases), GH13 (α-amylases), GH16 (β-1,4/β-1,3 glucanases), GH29 (α-fucosidases), GH42 (β-galactosidases), GH43 (β-D-xylosidases, α-L-arabinanases, α-L-arabinofuranosidase), GH71 (endo-1,3-α-glucosidases), and GH92 (α-mannosidases). Of the 12 of these proteins for which catalytic residues were known, at least 50% of the catalytic residues were present in all but one of the viral GHs, with six proteins containing all necessary residues for catalysis (one α-mannosidase, two α-fucosidases, one β-1,4-glucanase, one endo-β-1,4-mannosidase, and one β-galactosidase) (Supplementary Table 14). One GH5 representative was biochemically characterized in detail and exhibited hydrolysis of β-1,4 mannose linkages found in various substrates that include storage polysaccharides in plant roots and structural components of plant cell walls, including *Sphagnum* mosses indigenous to Stordalen Mire and particularly abundant in the bog habitat (Supplementary Fig. 10)[2]. Of the CAZy families sampled here, there were no prior viral representatives in the CAZy database for GH2, GH42, GH29, GH71, GH92, or GH13. Moreover, only three viral representatives out of ~9,000 GH5-affiliated sequences have been deposited, none of which had the enzymatic or structural characterization or the predicted mannanase activity identified here, and for GH16, there was only one viral representative with activity data in the CAZy database[3].

Our 14 viral GHs were found on 13 viral contigs, each from a different viral cluster (VC, approximately genus-level taxonomy[4]), suggesting no obvious signal for viral lineage conservation. Viral populations containing these GHs were detected in 34 of the 201 Stordalen Mire samples (6 palsa, 13 bog, and 15 fen), with coverages indicating the detection of a total of 272 genome copies for these viruses (73 in palsa, 100 in bog, and 99 in fen). We also searched for these GH PFAMs in two publicly available, large-scale viral datasets (the full Earth's virome

dataset[5], not to be confused with the soil-associated subset of this dataset used elsewhere in the manuscript, and global ocean viruses (GOV)[6]) and detected all but one of the GH PFAMs in Earth's virome but only two in GOV, perhaps suggesting that most of the identified GHs may be environment-specific, or at least uncommon in the oceans. Similar to our findings, a prior metagenomic study from switchgrass compost recovered a circular viral genome with a single GH43[7].

The Stordalen Mire viruses encoding most of these GHs had unidentified hosts (*n* = 9), but one virus with a GH was predicted to infect a member of the Betaproteobacteria, and three viruses (encoding four GHs) were predicted to infect Acidobacteria. The Acidobacteria are among the most abundant microbial lineages in Stordalen Mire, with a variety of complex carbon degradation capacities, so infection by GH-containing viruses could be consistent with supporting host metabolism throughout the infection cycle, analogous to photosynthesis genes in cyanophage (see below)[8].

With cyanophage photosynthesis genes as the classic example, "auxiliary metabolic genes" (AMGs) are virus-encoded genes with predicted functions in host metabolic pathways that are not directly related to virus-specific structures or functions. For instance, AMGs are generally not involved in host cell attachment and entry, viral replication, or viral structures[6, 9-13]. In the oceans, AMGs are generally predicted (and, in some cases, have been experimentally shown[14]) to keep their hosts healthy enough through the infection cycle to produce more viruses[13, 15]. These AMGs encode essential host metabolic genes that could be bottlenecks in host biochemical pathways[15]. However, the GHs encoded by Stordalen Mire viruses are generally predicted to degrade complex carbon commonly found in indigenous plant biomass, so, compared to marine AMGs, these viral GHs would not seem to have the same immediate effect on the infected host. We hypothesize that the Stordalen Mire viral GHs could do one or more of the following, all of which would have the effect of breaking down environmental or cell surface complex carbon into simple sugars:

1) serve to prime hosts in the immediate vicinity for the imminent "burst" of new viruses into the environment (*i.e.*, break down localized complex carbon to yield simple sugar substrates for nearby hosts to keep those hosts healthy for impending viral infections). Predicted Stordalen Mire viral GH activities included hydrolysis of mannan (confirmed biochemically), xyloglucan, α-arabinan, and pectin polymers, all of which are commonly found in nonvascular mosses (Bryophytes)[16], similar to those that dominate the vegetation at Stordalen Mire[17, 18]. We consider this scenario possible, given the predicted and demonstrated functional congruence between the viral GH target substrates and the available complex polymers in the sample site.

2) serve as a mechanism for bringing new functionality to the microbial hosts (*i.e.*, viruses would be conduits for transferring GH genes from host to host). Viruses are known agents of horizontal gene transfer and have been hypothesized to move other types of GHs from host to host[19].

3) serve the virus just prior to infection, for example, viral GHs have previously been hypothesized to function in biofilm degradation[20] and may increase host accessibility.

4) be part of host cell recognition, attachment, entry, and/or virion release (*e.g.*, via host capsule lipopolysaccharide and/or cell wall degradation)[21-23]. This explanation is consistent with prior studies that have shown that viruses encode GH genes for host entry and/or virion release, *e.g.*, lysozymes/endolysins and chitinases, and genes for peptidoglycan degradation[23-26]. Novel cell-entry functions for Stordalen Mire viral GHs may include GH43 and/or GH42 representatives that possibly target arabinogalactan in the cell walls of Mycobacteria (Actinobacteria)[27], or exo-acting α-fucosidases (GH29) that target terminal fucose-containing capsules[28]. However, the compositional polysaccharide knowledge of these host features are lacking, impeding specific linkages to these viral GHs.

Other putative AMGs

We also attempted to identify other putative AMGs in both the initial VirSorter-derived PFAM annotation and the newer annotation (see Methods), similar to previous manual annotation scans for microbial metabolic genes in viral genomic data[6, 29]. However, results were inconclusive (*i.e.*, putative functions could have been viral or may have been on regions of contigs that could not be unambiguously identified to be of viral origin).

Advantages of partial least squares (PLS) regression analyses

While standard regression assumes no error in predictor (X) variables, PLS regression (PLSR) may be considered a Type-II regression model, in that it does not assume zero error in predictor variables. A number of references that developed PLSR or advocate for its use[30-33] highlight the allowance for error in predictor variables as one of its positive features. For example, the PLSR tutorial of Geladi & Kowalski (1986) emphasizes that "measured data are never noise free"[30] and Wold et al. 2001 say that "the assumptions underlying PLS—correlations among the X's, noise in X, model errors—are more realistic than the MLR [multiple linear regression] assumptions of independent and error free X's".[33]

Supplementary References

1. Lombard, V., Golaconda Ramulu, H., Drula, E., Coutinho, P.M. & Henrissat, B. The carbohydrate-active enzymes database (CAZy) in 2013. *Nucleic Acids Research* **42**, D490-D495 (2014).
2. Kremer, C., Pettolino, F., Bacic, A. & Drinnan, A. Distribution of cell wall components in Sphagnum hyaline cells and in liverwort and hornwort elaters. *Planta* **219**, 1023-1035 (2004).
3. Sun, L., Gurnon, J.R., Adams, B.J., Graves, M.V. & Van Etten, J.L. Characterization of a β-1,3-Glucanase Encoded by Chlorella Virus PBCV-1. *Virology* **276**, 27-36 (2000).

4. Bolduc, B. et al. vConTACT: an iVirus tool to classify double-stranded DNA viruses that infect Archaea and Bacteria. *PeerJ* **5**, e3243 (2017).
5. Paez-Espino, D. et al. Uncovering Earth's virome. *Nature* **536**, 425-430 (2016).
6. Roux, S. et al. Ecogenomics and potential biogeochemical impacts of globally abundant ocean viruses. *Nature* **537**, 689-693 (2016).
7. Allgaier, M. et al. Targeted Discovery of Glycoside Hydrolases from a Switchgrass-Adapted Compost Community. *PLOS ONE* **5**, e8812 (2010).
8. Sullivan, M.B. et al. Prevalence and Evolution of Core Photosystem II Genes in Marine Cyanobacterial Viruses and Their Hosts. *PLOS Biology* **4**, e234 (2006).
9. Sullivan, M.B. et al. Prevalence and evolution of core photosystem II genes in marine cyanobacterial viruses and their hosts. *PLoS Biol* **4** (2006).
10. Brum, J.R. & Sullivan, M.B. Rising to the challenge: accelerated pace of discovery transforms marine virology. *Nat Rev Micro* **13**, 147-159 (2015).
11. Anantharaman, K. et al. Sulfur Oxidation Genes in Diverse Deep-Sea Viruses. *Science* **344**, 757-760 (2014).
12. Hurwitz, B.L., Hallam, S.J. & Sullivan, M.B. Metabolic reprogramming by viruses in the sunlit and dark ocean. *Genome Biology* **14**, R123 (2013).
13. Bragg, J.G. & Chisholm, S.W. Modelling the fitness consequences of a cyanophage-encoded photosynthesis gene. *PLoS One* **3** (2008).
14. Lindell, D., Jaffe, J.D., Johnson, Z.I., Church, G.M. & Chisholm, S.W. Photosynthesis genes in marine viruses yield proteins during host infection. *Nature* **438** (2005).
15. Thompson, L.R. et al. Phage auxiliary metabolic genes and the redirection of cyanobacterial host carbon metabolism. *Proceedings of the National Academy of Sciences* **108**, 757-764 (2011).
16. Moller, I. et al. High-throughput mapping of cell-wall polymers within and between plants using novel microarrays. *The Plant Journal* **50**, 1118-1128 (2007).
17. Hodgkins, S.B. et al. Changes in peat chemistry associated with permafrost thaw increase greenhouse gas production. *Proceedings of the National Academy of Sciences* **111**, 5819-5824 (2014).
18. McCalley, C.K. et al. Methane dynamics regulated by microbial community response to permafrost thaw. *Nature* **514**, 478-481 (2014).
19. Strachan, C.R. et al. Metagenomic scaffolds enable combinatorial lignin transformation. *Proceedings of the National Academy of Sciences* **111**, 10143-10148 (2014).
20. Maaroufi, H. & Levesque, R.C. Glycoside hydrolase family 32 is present in Bacillus subtilis phages. *Virology Journal* **12**, 157 (2015).
21. Byl, C.V. & Kropinski, A.M. Sequence of the Genome of Salmonella Bacteriophage P22. *Journal of Bacteriology* **182**, 6472-6481 (2000).
22. Hynes, W.L. & Ferretti, J.J. Sequence analysis and expression in Escherichia coli of the hyaluronidase gene of Streptococcus pyogenes bacteriophage H4489A. *Infection and Immunity* **57**, 533-539 (1989).

23. Davison, M., Treangen, T.J., Koren, S., Pop, M. & Bhaya, D. Diversity in a Polymicrobial Community Revealed by Analysis of Viromes, Endolysins and CRISPR Spacers. *PLOS ONE* **11**, e0160574 (2016).
24. Davies, G. & Henrissat, B. Structures and mechanisms of glycosyl hydrolases. *Structure* **3**, 853-859.
25. Ubhayasekera, W. Structure and function of chitinases from glycoside hydrolase family 19. *Polymer International* **60**, 890-896 (2011).
26. Yuan, Y. & Gao, M. Proteomic Analysis of a Novel Bacillus Jumbo Phage Revealing Glycoside Hydrolase As Structural Component. *Frontiers in Microbiology* **7** (2016).
27. Wu, Y., Xiong, D.-C., Chen, S.-C., Wang, Y.-S. & Ye, X.-S. Total synthesis of mycobacterial arabinogalactan containing 92 monosaccharide units. *Nature Communications* **8**, 14851 (2017).
28. Wu, J.H. et al. Contribution of Fucose-Containing Capsules in Klebsiella pneumoniae to Bacterial Virulence in Mice. *Experimental Biology and Medicine* **233**, 64-70 (2008).
29. Roux, S., Hallam, S.J., Woyke, T. & Sullivan, M.B. Viral dark matter and virus–host interactions resolved from publicly available microbial genomes. *eLife* **4**, e08490 (2015).
30. Geladi, P. & Kowalski, B.R. Partial least-squares regression: a tutorial. *Analytica Chimica Acta* **185**, 1-17 (1986).
31. Wold, S., Ruhe, A., Wold, H. & Dunn III, W.J. *SIAM Journal on Scientific and Statistical Computing* **5**, 735-743 (1984).
32. Høy, M., Steen, K. & Martens, H. Review of partial least squares regression prediction error in Unscrambler. *Chemometrics and Intelligent Laboratory Systems* **44**, 123-133 (1998).
33. Wold, S., Sjöström, M. & Eriksson, L. PLS-regression: a basic tool of chemometrics. *Chemometrics and Intelligent Laboratory Systems* **58**, 109-130 (2001).

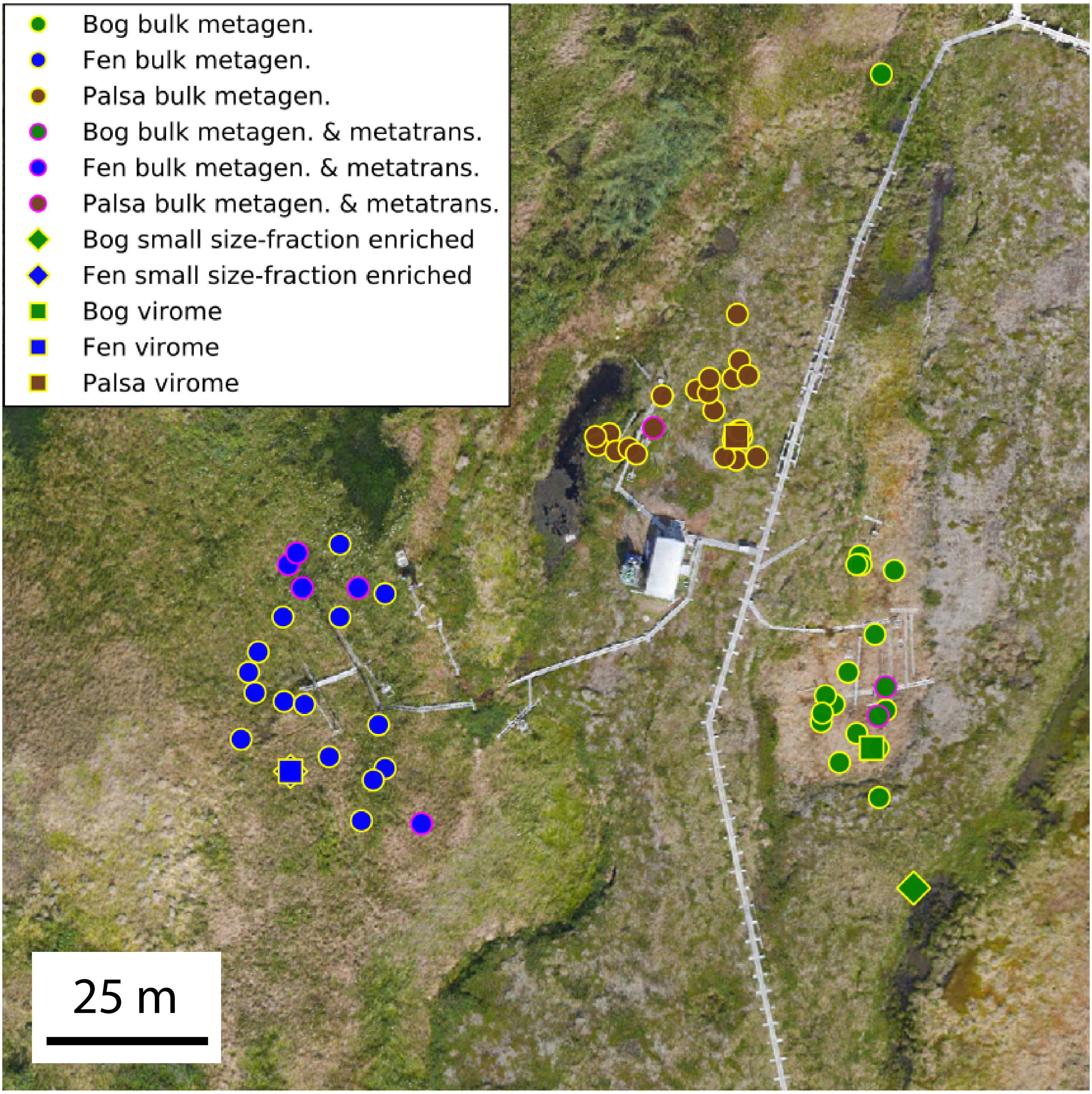

Supplementary Figure 1: Metagenomic and metatranscriptomic sampling locations
Aerial view of metagenomic (both yellow and pink outlines) and metatranscriptomic (pink outlines only) sampling locations at Stordalen Mire near Abisko, Sweden. Metagenomic samples are color-coded by habitat (palsa = brown, bog = green, fen = blue). Bulk soil metagenomes used for ecological and statistical analyses are indicated by circles (both yellow and pink outlines). Other metagenomes (rhombuses and squares) were used only to improve the database of viral populations that could have been detected in the bulk soil metagenomes. The underlying image was collected via drone and extensively manually curated for GPS accuracy. Sampling locations were mapped onto this image based on their GPS coordinates, as in Supplementary Table 1. Bulk = bulk soil, metagen. = metagenomes, metatrans. = metatranscriptomes, small size-fraction enriched = small size-fraction enriched metagenomes, virome = viral size-fraction metagenomes.

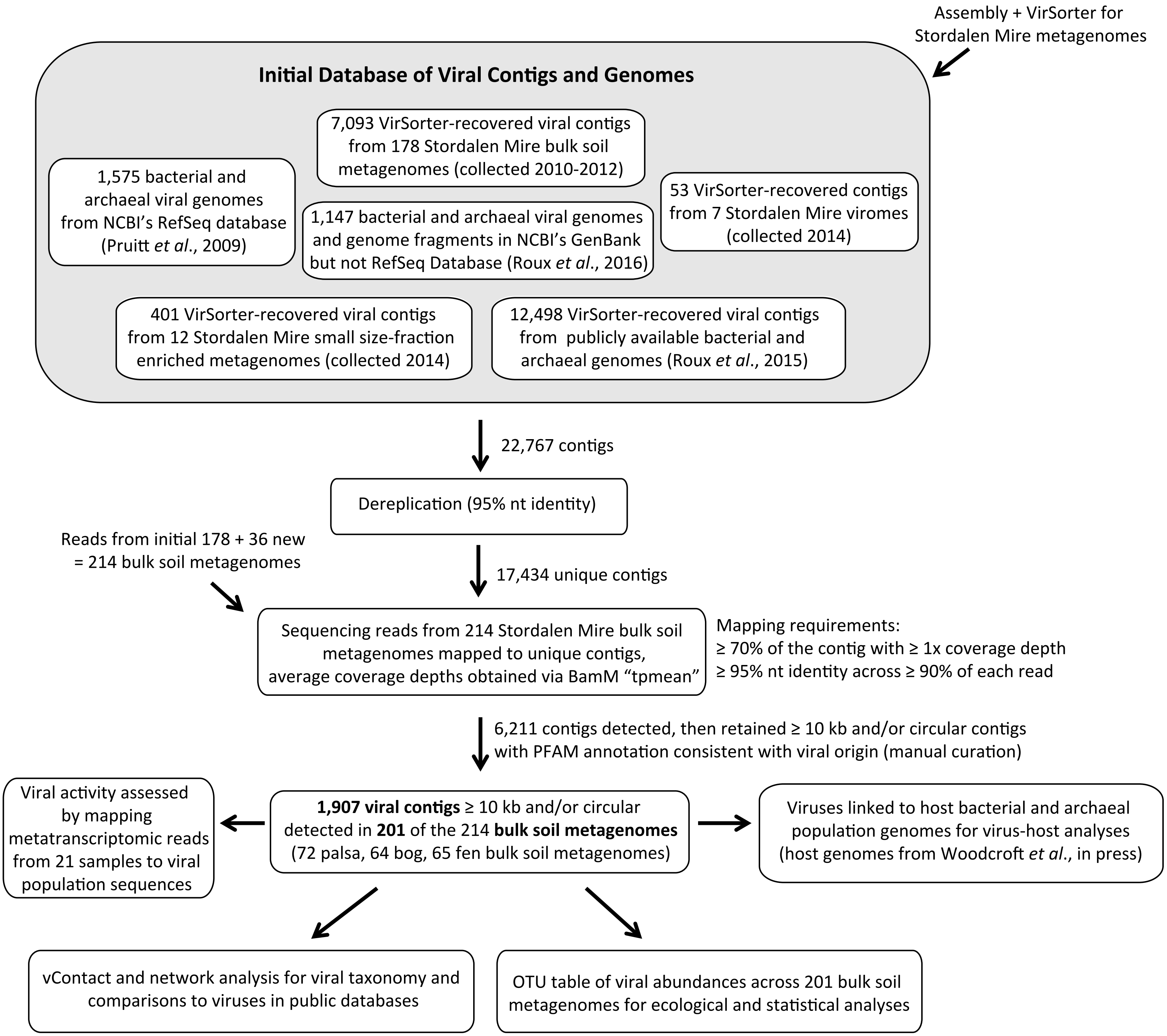

Supplementary Figure 2: Overview of Bioinformatic Processing

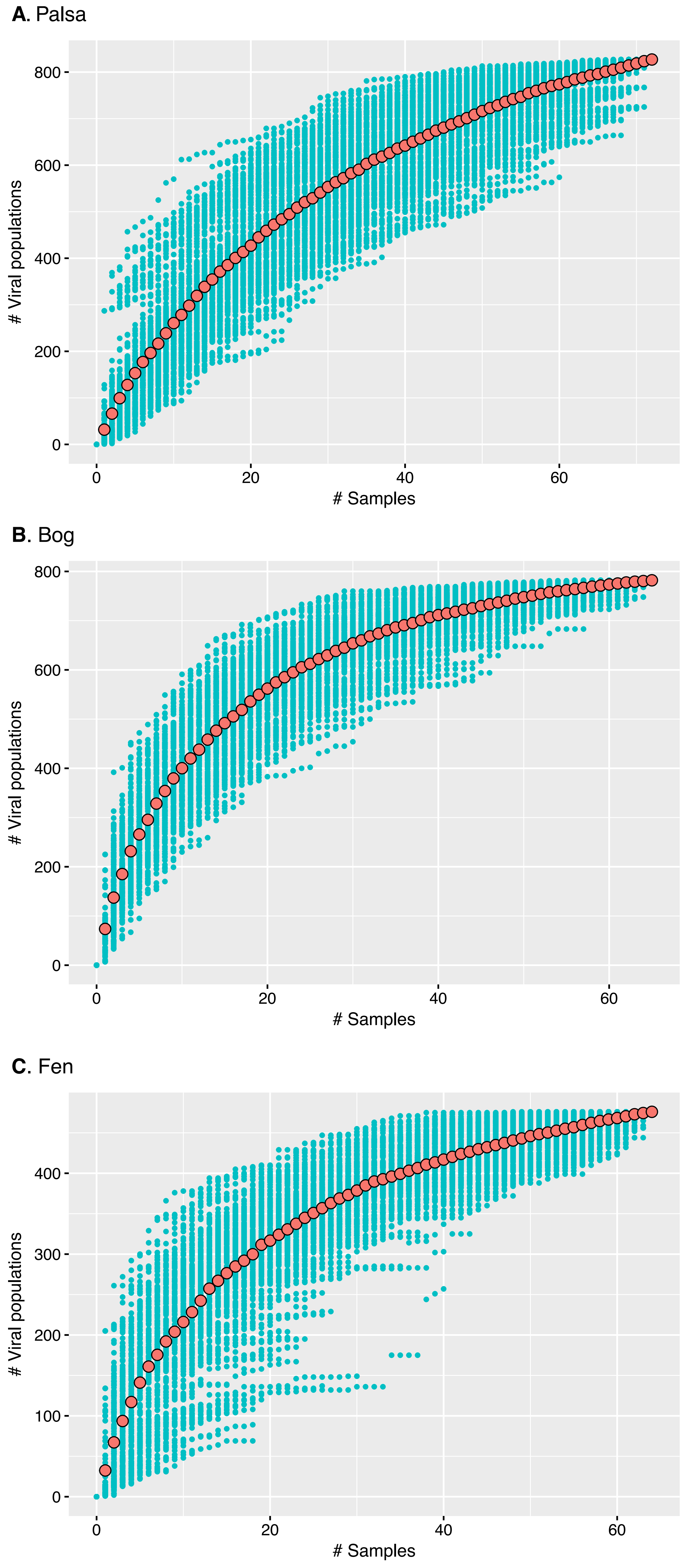

Supplementary Figure 3: Accumulation curves for Stordalen Mire viral populations separated by habitat

Accumulation curve of viral populations. Teal traces represent 200 iterations (sample order randomizations), and red points are means. **A**. Palsa samples (*n*=72), **B**. Bog samples (*n*=65), and **C**. Fen samples (*n*=64).

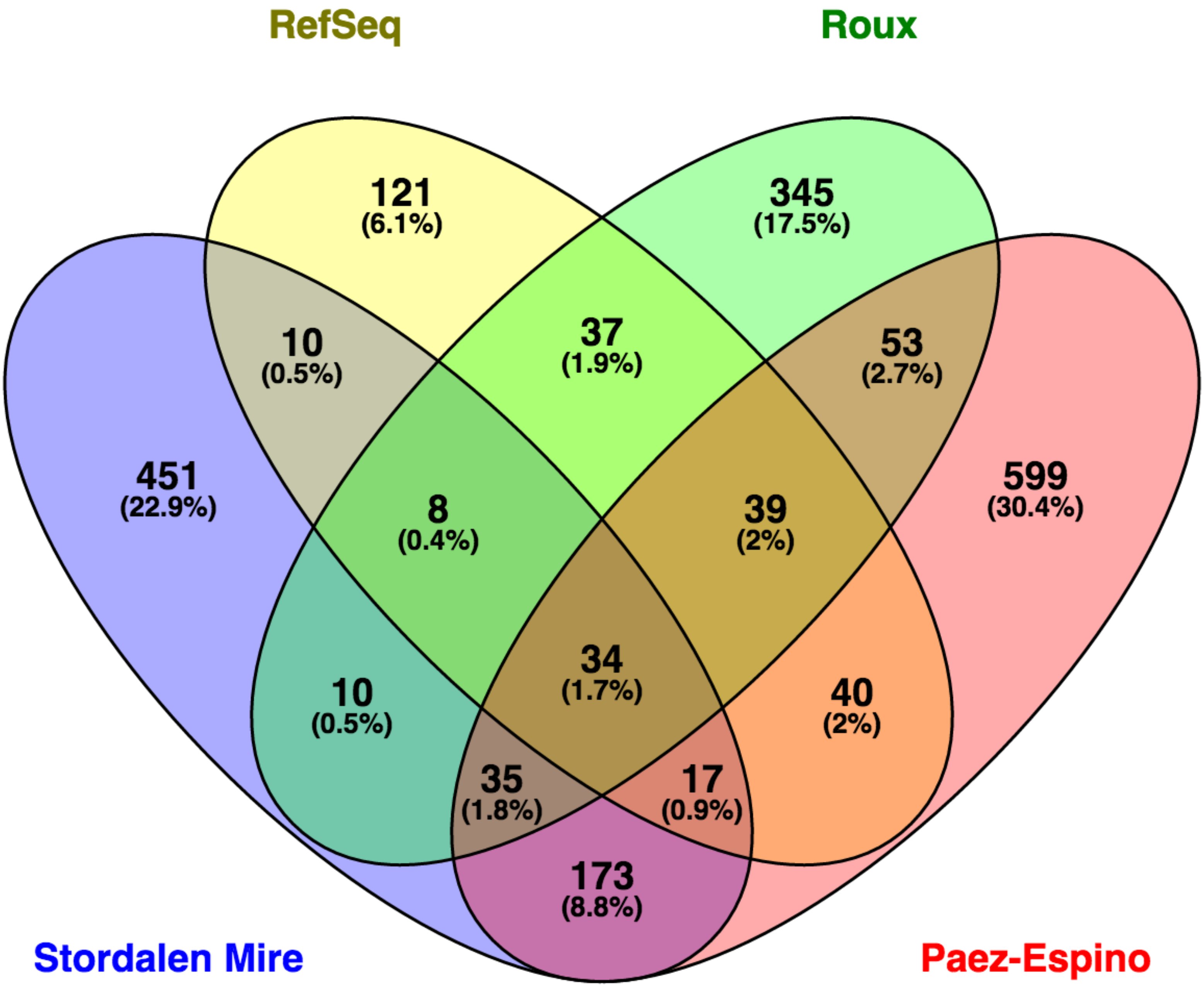

Supplementary Figure 4: Viral clusters within and across datasets

Venn diagram of 1,972 viral clusters (VCs) within and across four datasets: Stordalen Mire (this study), RefSeq (prokaryotic viral genome sequences from NCBI's RefSeq v75), Roux (soil-associated viral contigs >10 kb from a dataset of viral contigs mined from microbial isolate genomes), and Paez-Espino (soil-associated viral contigs >10 kb from a dataset of viral contigs mined from bulk metagenomes); VCs represent approximately genus-level taxonomy and are groups of genomes and contigs clustered based on shared predicted protein content. Numbers and percentages represent the total number and percent, respectively, of VCs shared among the dataset(s) in a given section of the diagram.

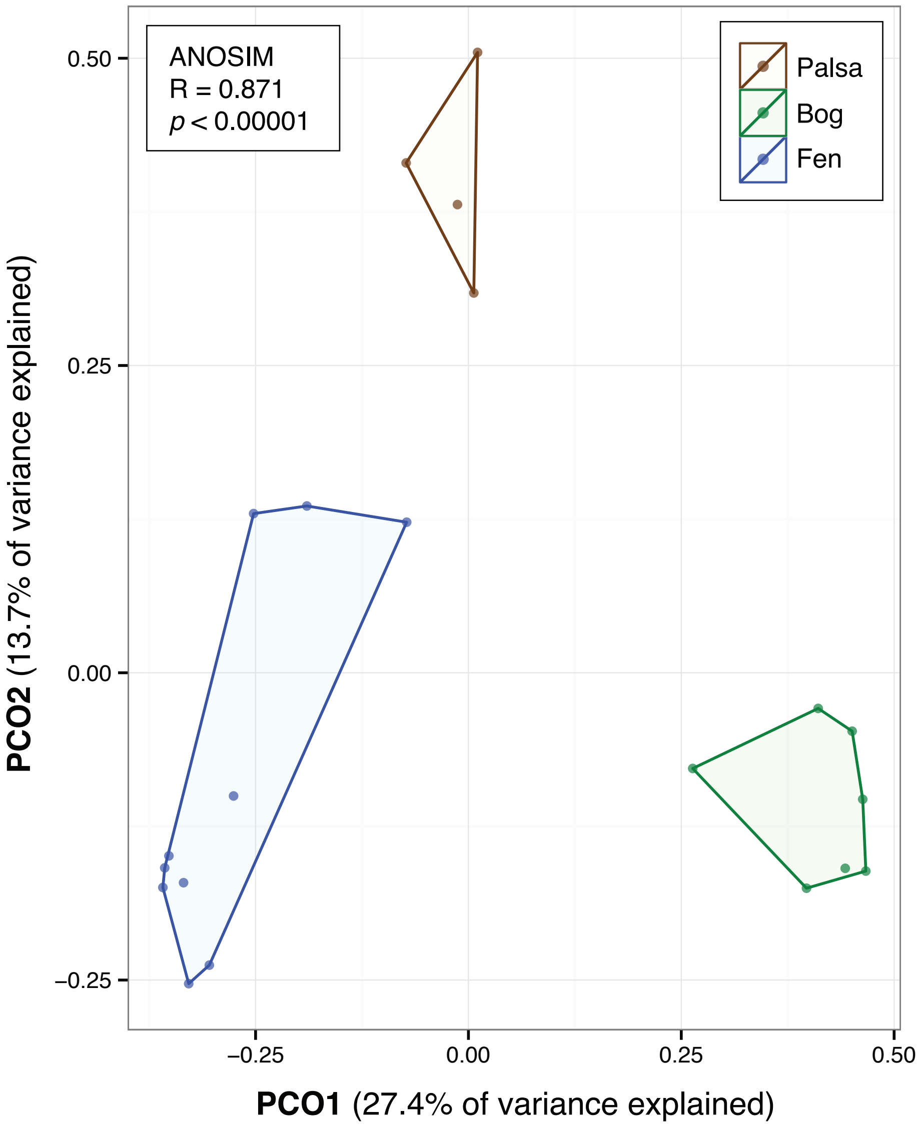

Supplementary Figure 5: Viral populations detected in metatranscriptomes
Principal coordinates analysis (PCoA) of "active" viral community composition, as estimated from metatranscriptomic read mapping to viral population sequences and Bray-Curtis dissimilarities (calculated for viral populations detected in at least 2 metatranscriptomes (n = 665 of 1,907)); each point is one sample (n=21), and the proximity of points indicates similarity in "active" viral community composition; ANOSIM statistics indicate the extent to which "active" viral community composition differs by habitat.

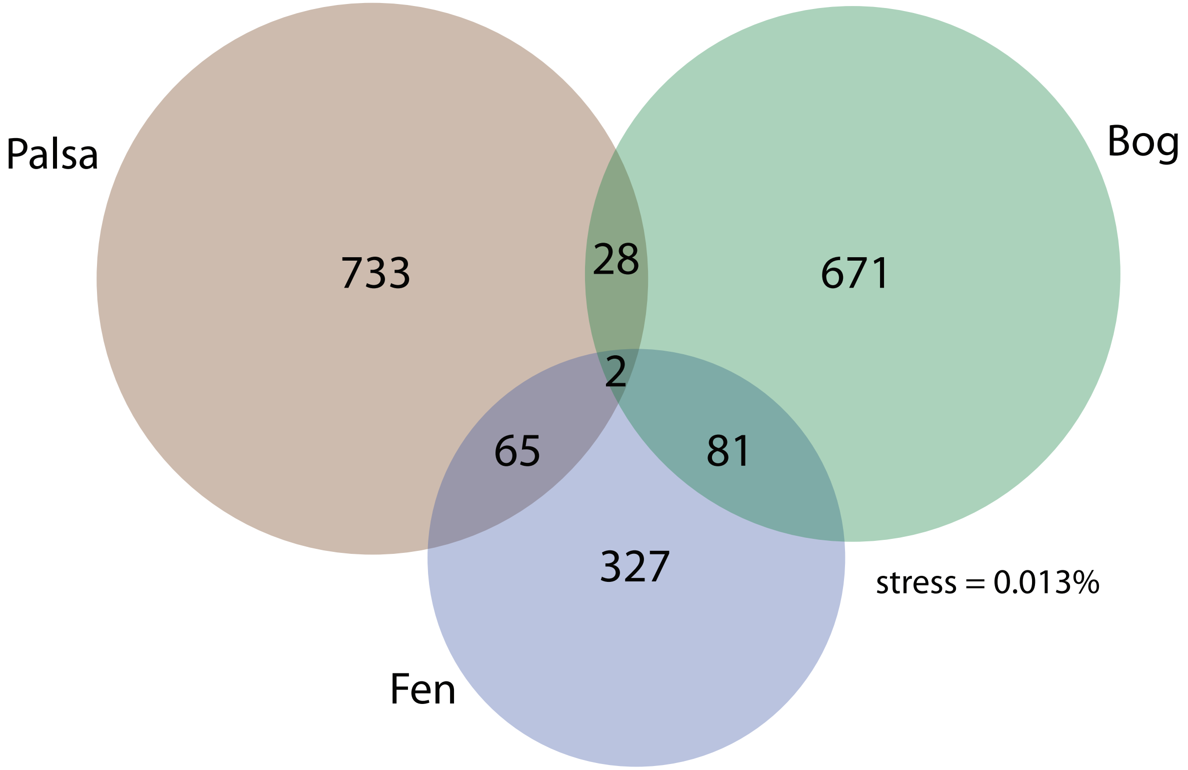

Supplementary Figure 6: Viral populations detected in each habitat
Euler diagram depicting unique and shared viral populations (*n*=1,907) across the three habitats (palsa, bog, and fen), based on populations detected (presence-absence) in each sample (*n*=201) through metagenomic read mapping to viral contigs; the stress value indicates the extent to which the diagrammatic representation recapitulates the data, with a stress of 0% indicating no distortion of data in the diagram.

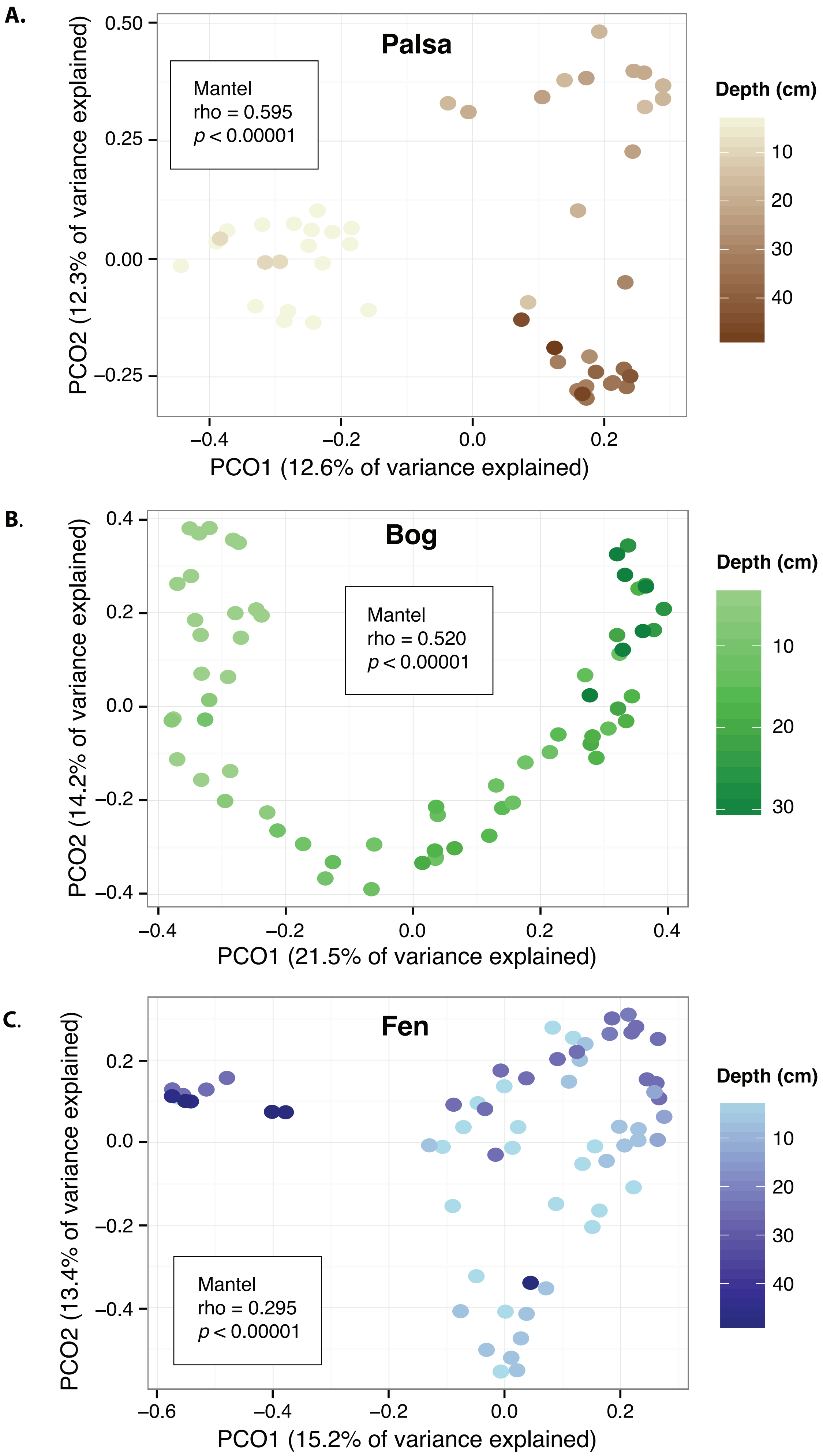

Supplementary Figure 7: Viral community composition within habitats by depth
Principal coordinates analysis (PCoA) of viral community composition, as derived from read mapping to viral contigs (*n*=1,907) and Bray-Curtis dissimilarities; each point is one sample, and the proximity of points indicates similarity in viral community composition; points are colored by the depth below the surface from which the core sample was recovered; Mantel correlation statistics indicate the extent to which viral community composition in a given habitat was correlated with depth. **A**. Palsa samples (*n*=72), **B**. Bog samples (*n*=65), and **C**. Fen samples (*n*=64).

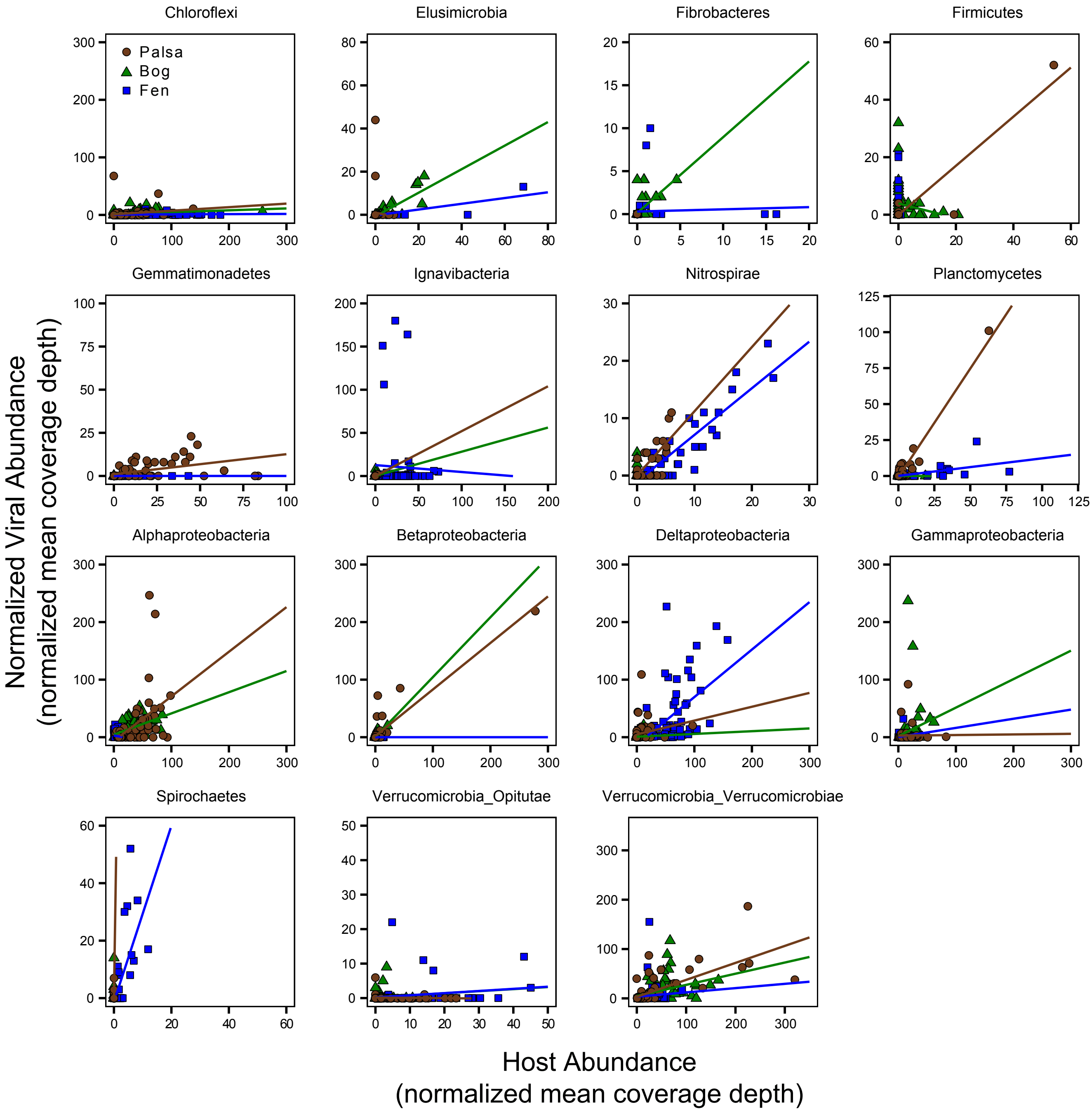
Chloroflexi
Elusimicrobia
Fibrobacteres
Firmicutes
Gemmatimonadetes
Ignavibacteria
Nitrospirae
Planctomycetes
Alphaproteobacteria
Betaproteobacteria
Deltaproteobacteria
Gammaproteobacteria
Spirochaetes
Verrucomicrobia_Opitutae
Verrucomicrobia_Verrucomicrobiae
Palsa
Bog
Fen
Normalized Viral Abundance
(normalized mean coverage depth)
Host Abundance
(normalized mean coverage depth)

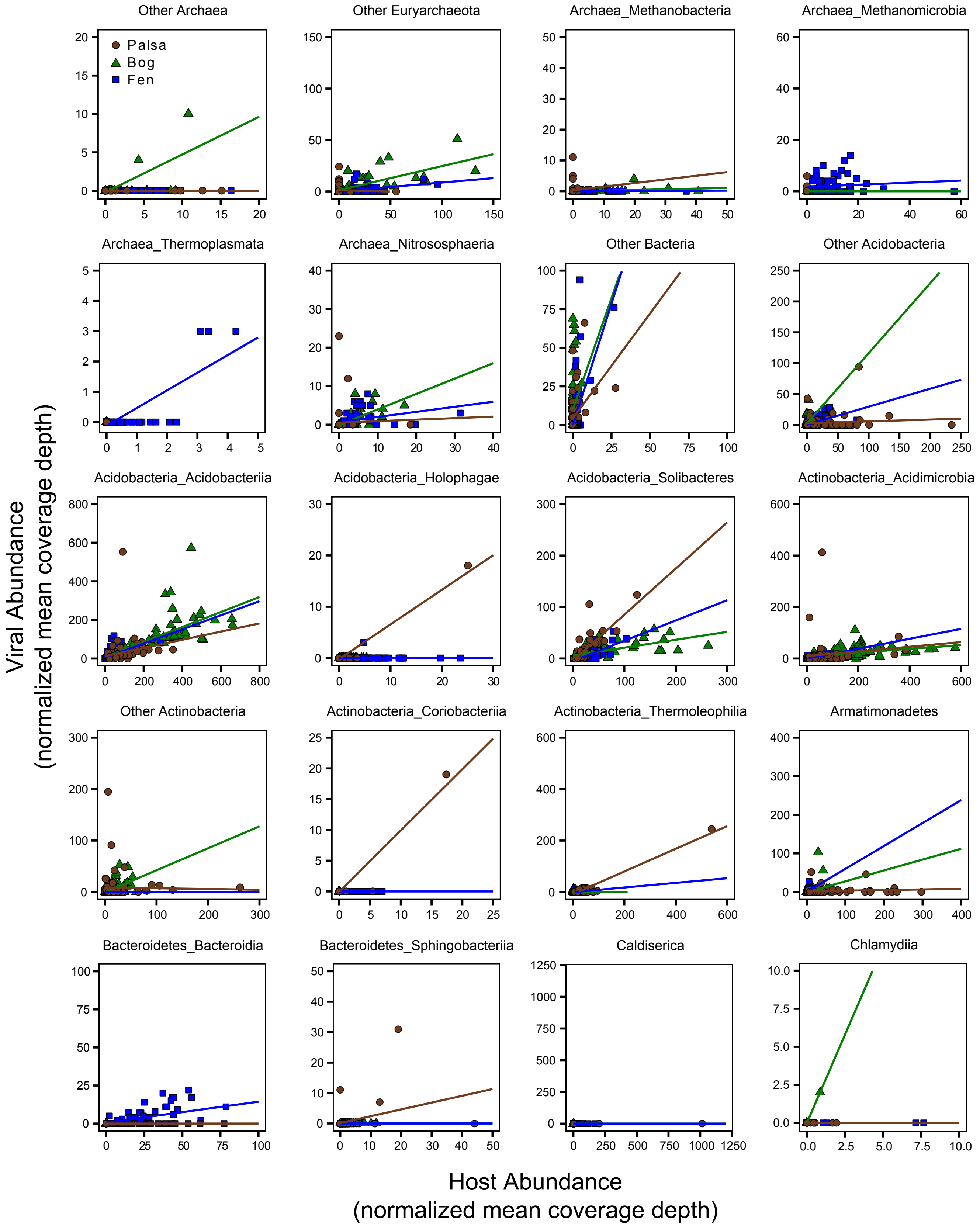
Other Archaea
Palsa
Bog
Fen
Other Euryarchaeota
Archaea_Methanobacteria
Archaea_Methanomicrobia
Archaea_Thermoplasmata
Archaea_Nitrososphaeria
Other Bacteria
Other Acidobacteria
Acidobacteria_Acidobacteriia
Acidobacteria_Holophagae
Acidobacteria_Solibacteres
Actinobacteria_Acidimicrobia
Other Actinobacteria
Actinobacteria_Coriobacteriia
Actinobacteria_Thermoleophilia
Armatimonadetes
Bacteroidetes_Bacteroidia
Bacteroidetes_Sphingobacteriia
Caldiserica
Chlamydiia
Viral Abundance
(normalized mean coverage depth)
Host Abundance
(normalized mean coverage depth)

Supplementary Figure 8: Relationships between lineage-specific virus-host abundances across the thaw gradient (previous two pages)

The host lineage is indicated at the top of each plot. Host lineage abundance (x-axis) and the abundance of viruses (y-axis) for that host (both calculated as the mean coverage depth from metagenomic read mapping, normalized by the number of reads in the sample) are plotted for each sample. Note different axis maxima among graphs. Color-coded linear regression lines for each habitat are presented (no line for a particular habitat indicates insufficient data). Linear regression and ANOVA statistics for virus-host abundances across the thaw gradient for each host lineage are in Table S9. Palsa n=72, bog n= 65, fen n=64.

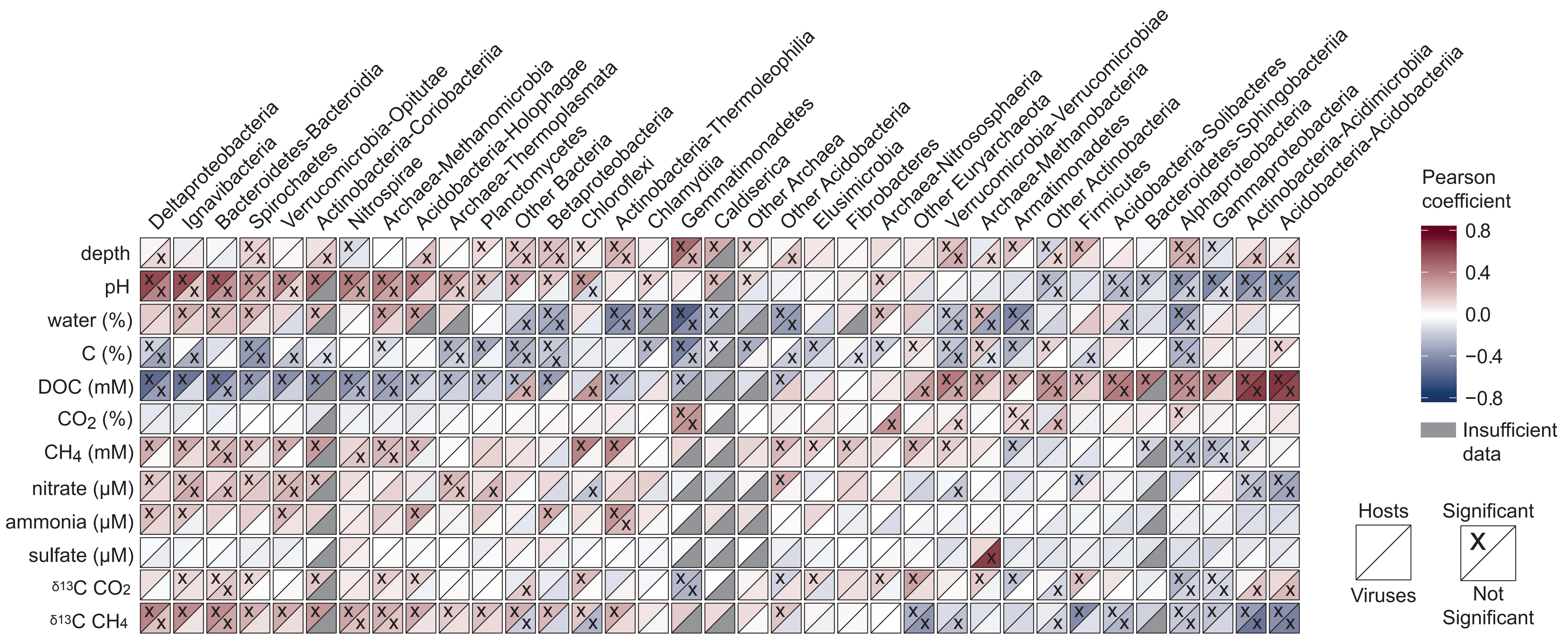

Supplementary Figure 9: Correlations of virus and host abundances with geochemical and environmental variables by host lineage

Pearson's correlation coefficients for host lineages (tops of boxes) and their viruses (bottoms of boxes), correlated with environmental and geochemical measurements (significant when $p < 0.05$, values appear in Supplementary Table 12). Environmental and geochemical variables are in rows and host lineages are in columns. Pearson's correlation coefficients from 294 significantly correlated abundances ($p < 0.05$) are depicted with an "x"; the probability of observing only 53 or more such $p < 0.05$ correlations given 840 tests is less than 5% under the null hypothesis.

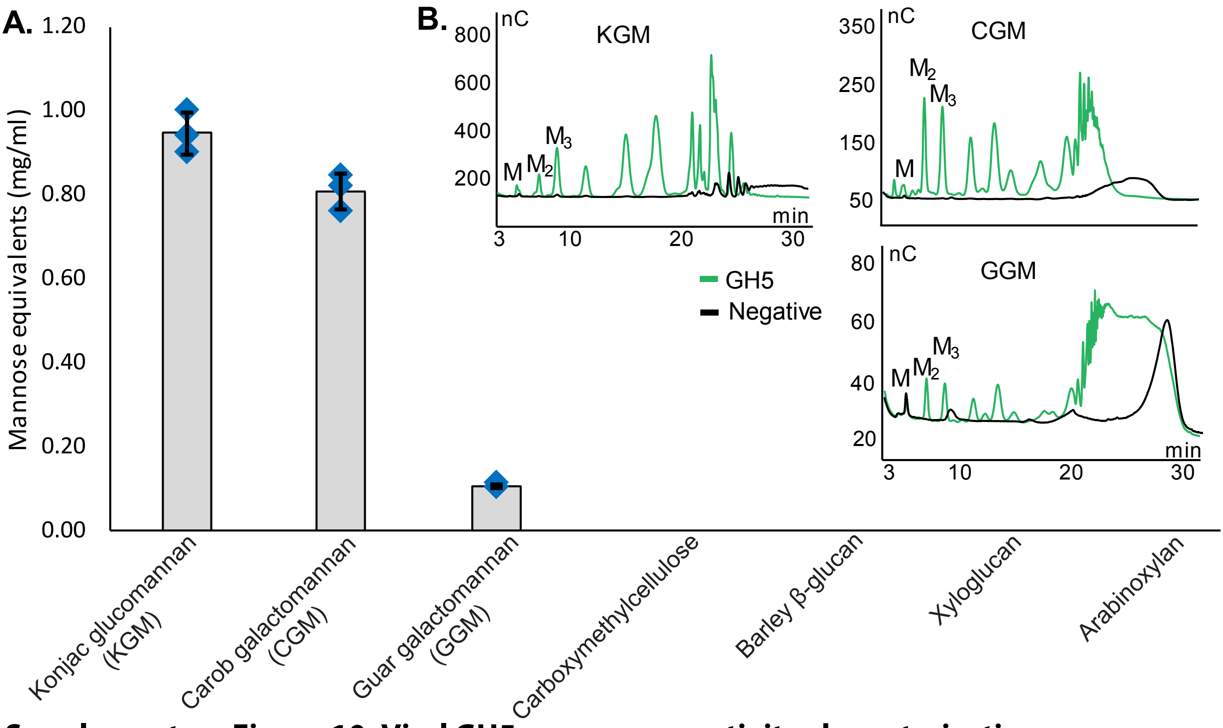

Supplementary Figure 10: Viral GH5 mannanase activity characterization

A. Degradation of soluble polysaccharides by the GH5 enzyme, measured as reducing ends (mannose equivalents) recovered. Assays were performed in triplicate at 40 °C with 5 mg/ml substrate, 20 mM citrate buffer pH 4.0 (similar to sample site) and 0.5 µM enzyme for one hour. Reducing ends were quantified by the DNS-method against a standard curve of mannose. Error bars represent standard deviations among three replicates, individual values are indicated by blue diamonds. The mean values for KGM, CGM, and GGM were 0.936 mg/ml, 0.810 mg/ml, and 0.108 mg/ml, respectively. The three substrates that yielded activity contain β-1,4 mannose linkages. No activity was exhibited on cellulose (β-1,4 linked glucose), mixed-linkage glucans (β-1,4/β-1,3 linked glucose), xyloglucans (β-1,4 linked glucose), or arabinoxylans (β-1,4 linked xylose).

B. Degradation products from soluble mannan polysaccharides by the GH5 enzyme, analyzed by high-pH anion-exchange chromatography–pulsed amperometric detection (HPAEC-PAD) in triplicate. Chromatogram x-axes: retention time (min), y-axes: signal strength (nC). Identified peaks depicting mannose (M) and -1,4-mannooligosaccharides (M2-3) are annotated with their degree of polymerization (DP) as subscripts. Assay conditions were as described above, with an extended incubation of 18 hours. Reactions were stopped by addition of NaOH to a final concentration of 0.1M. KGM: Konjac glucomannan; CGM: Carob galactomannan; GGM: Guar galactomannan.
