# Supplementary material for: Host-linked soil viral ecology along a permafrost thaw gradient
Source: Nat Microbiol. 2018 Jul 16;3(8):870–80. doi: 10.1038/s41564-018-0190-y (PMC6786970; doi:10.1038/s41564-018-0190-y)
Supplement: Supplementary file 2 — Reporting Summary [file 41564_2018_190_MOESM2_ESM.pdf]

nature research

Corresponding author(s): Matthew B. Sullivan

Reporting Summary

Nature Research wishes to improve the reproducibility of the work that we publish. This form provides structure for consistency and transparency in reporting. For further information on Nature Research policies, see Authors & Referees and the Editorial Policy Checklist.

Statistical parameters

When statistical analyses are reported, confirm that the following items are present in the relevant location (e.g. figure legend, table legend, main text, or Methods section).

n/a	Confirmed	
☐	☒	The exact sample size (n) for each experimental group/condition, given as a discrete number and unit of measurement
☐	☒	An indication of whether measurements were taken from distinct samples or whether the same sample was measured repeatedly
☐	☒	The statistical test(s) used AND whether they are one- or two-sided<br>*Only common tests should be described solely by name; describe more complex techniques in the Methods section.*
☐	☒	A description of all covariates tested
☐	☒	A description of any assumptions or corrections, such as tests of normality and adjustment for multiple comparisons
☐	☒	A full description of the statistics including central tendency (e.g. means) or other basic estimates (e.g. regression coefficient) AND variation (e.g. standard deviation) or associated estimates of uncertainty (e.g. confidence intervals)
☐	☒	For null hypothesis testing, the test statistic (e.g. F, t, r) with confidence intervals, effect sizes, degrees of freedom and P value noted<br>*Give P values as exact values whenever suitable.*
☒	☐	For Bayesian analysis, information on the choice of priors and Markov chain Monte Carlo settings
☒	☐	For hierarchical and complex designs, identification of the appropriate level for tests and full reporting of outcomes
☒	☐	Estimates of effect sizes (e.g. Cohen's d, Pearson's r), indicating how they were calculated
☐	☒	Clearly defined error bars<br>*State explicitly what error bars represent (e.g. SD, SE, CI)*

Our web collection on statistics for biologists may be useful.

Software and code

Policy information about availability of computer code

Data collection	This information is provided in the methods section. There are too many software programs to list here, but all are publicly available, either through prior publications or as indicated in the methods section.
Data analysis	This information is provided in the methods section. There are too many software programs to list here, but all are publicly available, either through prior publications or as indicated in the methods section.

For manuscripts utilizing custom algorithms or software that are central to the research but not yet described in published literature, software must be made available to editors/reviewers upon request. We strongly encourage code deposition in a community repository (e.g. GitHub). See the Nature Research guidelines for submitting code & software for further information.

Data

Policy information about availability of data

All manuscripts must include a data availability statement. This statement should provide the following information, where applicable:

- Accession codes, unique identifiers, or web links for publicly available datasets
- A list of figures that have associated raw data
- A description of any restrictions on data availability

Sequencing data described in this manuscript, including metagenomic and metatranscriptomic data also described in Woodcroft et al. in review14, are submitted

under NCBI BioProject accession number PRJNA386568. Within that BioProject, the 1,907 viral population contig sequences have been deposited at DDBJ/ENA/GenBank under accession number QGNH00000000. The version described in this paper is version QGNH01000000. The Supplementary Information includes 15 supplementary datasets (Supplementary Tables 1-15). Other data that support the findings of this study are available from the corresponding author upon request.

Field-specific reporting

Please select the best fit for your research. If you are not sure, read the appropriate sections before making your selection.

☒ Life sciences ☐ Behavioural & social sciences ☐ Ecological, evolutionary & environmental sciences

For a reference copy of the document with all sections, see nature.com/authors/policies/ReportingSummary-flat.pdf

Life sciences study design

All studies must disclose on these points even when the disclosure is negative.

Sample size	As stated in the Methods section, sample size was not predetermined.
Data exclusions	No data that met standard criteria were excluded (see methods for thresholds, summarized in Supplementary Figure 2).
Replication	There were no experiments per se, but the three habitats were sampled repeatedly (> 50 samples per habitat, see methods), and these samples can be considered biological replicates.
Randomization	Not relevant; there were no experiments related to the key findings
Blinding	Investigators were not blinded to sample allocation, but statistical methods were used to determine whether categorical criteria (e.g., habitat) better predicted community composition than random assignment of samples to habitats

Reporting for specific materials, systems and methods

Materials & experimental systems

n/a	Involved in the study	
☒	☐	Unique biological materials
☒	☐	Antibodies
☒	☐	Eukaryotic cell lines
☒	☐	Palaeontology
☒	☐	Animals and other organisms
☒	☐	Human research participants

Methods

n/a	Involved in the study	
☒	☐	ChIP-seq
☒	☐	Flow cytometry
☒	☐	MRI-based neuroimaging
